# The pterocarpan (+)-PTC modulates cytoskeletal proteins and induces apoptosis in metastatic castration-resistant prostate cancer: a proteomic perspective

**DOI:** 10.3389/fphar.2026.1770249

**Published:** 2026-06-03

**Authors:** José de Brito Vieira Neto, Kaio Moraes de Farias, Sarah Leyenne Alves Sales, Vitoria Braga Melo, Stella Maria Nascimento Macêdo, Carlos Roberto Koscky Paier, Maria Júlia Bezerra, Arlindo A. Moura, Hernandes F. Carvalho, Felipe Domingos de Sousa, Adriano Aquino, Ana Cristina de Oliveira Monteiro, Daniel Martins-de-Souza, Martin G. Banwell, Cristiana Libardi Miranda Furtado, Claudia Pessoa

**Affiliations:** 1 Drug Research and Development Center, Department of Physiology and Pharmacology, Federal University of Ceará, Fortaleza, Brazil; 2 Programa de Pós-Graduação em Biotecnologia, Rede Nordeste de Biotecnologia, Federal University of Ceará, Fortaleza, Brazil; 3 Drug Research and Development Center, Postgraduate Program in Translational Medicine, Federal University of Ceará, Fortaleza, Brazil; 4 Department of Animal Science, Federal University of Ceará, Fortaleza, Ceará, Brazil; 5 Department of Structural and Functional Biology, Biology Institute, State University of Campinas, Campinas, Brazil; 6 Center for Experimental Biology, Universidade de Fortaleza, Fortaleza, Brazil; 7 Laboratory of Neuroproteomics, Department of Biochemistry and Tissue Biology, Institute of Biology, University of Campinas, Campinas, Brazil; 8 Graduate Program in Medical Sciences, Experimental Biology Center, University of Fortaleza, Fortaleza, Brazil; 9 Experimental Medicine Research Cluster (EMRC), University of Campinas, Campinas, Brazil; 10 D'Or Institute for Research and Education (IDOR), SãoPaulo, Brazil; 11 INCT in Modelling Human Complex Diseases With 3D Platforms (Model3D), Conselho Nacional de Desenvolvimento Científico e Tecnológico, SãoPaulo, Brazil; 12 Institute for Advanced and Applied Chemical Synthesis, Jinan University, Zhuhai, China; 13 Department of Genetics, Ecology and Evolution, Institute of Biological Sciences, Federal University of Minas Gerais, Belo Horizonte, Brazil

**Keywords:** apoptosis, cytoskeleton proteins, prostate cancer, proteomics, pterocarpans

## Abstract

Treatment of metastatic, castration-resistant prostate cancer (mCRPC) remains clinically challenging due to tumor heterogeneity and resistance to standard microtubule-targeting agents, such as docetaxel and cabazitaxel. The natural pterocarpan (+)-(6aS,11aS)-2,3,9-trimethoxypterocarpan [(+)-PTC] has previously shown selective cytotoxicity and disruption of bipolar spindle assembly in mCRPC PC-3 cells yet its full mechanism of action and global proteomic impact remain uncharacterized. PC-3 cells were treated with (+)-PTC (2.0 µM or 8.0 µM) for 24 h and assessed by flow cytometry (Annexin V and rhodamine 123) Western blot, and caspase-3/7 luminescence assays. Global proteomic profiling was performed by quantitative LC-MS/MS using a 2D nano-UPLC data-independent acquisition platform with nocodazole (0.25 µM, microtubule depolymerizer) and monastrol (50 µM, Eg5 inhibitor) as comparators. (+)-PTC-induced concentration-dependent apoptosis (19.8% early apoptotic cells at 2.0 µM; 26.1% at 8.0 µM) and progressive mitochondrial membrane depolarization. Western blot and caspase activities assays confirmed upregulation of BAX and caspase-7, consistent with intrinsic pathway activation. Proteomic analysis identified 212 differentially expressed proteins (DEPs) in (+)-PTC-treated cells, with over 80% overlap with nocodazole-treated cells (82% among upregulated and 84% among downregulated proteins), while clustering with monastrol was markedly lower. The top upregulated proteins, TTLL3, ANAPC7, PIK3CA, ARID4B, and COL16A1, are linked to microtubule dynamics and cell cycle regulation; the main downregulated, namely KDM2B, PTOV1, YWHAQ, PSMB6, and PRKCB, are involved in cell survival, protein homeostasis and mitotic checkpoint control. These findings provide proteomic evidence that (+)-PTC interferes with cytoskeletal protein dynamics and promotes apoptosis in mCRPC and so warranting its further investigation as a candidate anti-cancer scaffold.

## 1 Introduction

Prostate cancer (PC) is the second most common type of cancer in men, surpassed only by lung cancer. This public health problem is widely recognized, with almost 400,000 deaths worldwide per year and more than one million new cases diagnosed world-wide in 2020 alone (Sung et al., 2021). The high incidence and prevalence of prostate cancer is evident in a multitude of countries and so highlighting the global impact of this condition (Sung et al., 2021; Wang et al., 2022). Although potentially curable when confined to the prostate, about 20% of patients will present with metastases, this being the leading cause of PC-related deaths. Additionally, some individuals will experience disease progression despite the application of treatments such as surgery, radiotherapy and/or androgen-deprivation therapy (ADT) (Klusa et al., 2020; Klusa et al., 2020). While prostate cancer responds well to initial androgen deprivation, it often progresses to an androgen-non-responsive phenotype after treatment and so leading to a stage called castration-resistant prostate cancer or metastatic; castration-resistant prostate cancer (mCRPC) (Harris et al., 2009; Karantanos et al., 2015; Katzenwadel and Wolf, 2015). Effective targeted treatments for mCRPC are not currently available due to the heterogeneous nature of the disease, the complexity of the associated molecular pathways and emerging drug-resistance to various anti-tumor agents (Mokhtari et al., 2017; Wasim et al., 2022).

Natural products, which offer great structural diversity, have been a tremendous source of new drugs or drug-leads (Huang et al., 2021). One of the breakthroughs in the search for natural anti-cancer agents has been the discovery of secondary metabolites that interfere with the lengthening (polymerisation) and/or shortening (depolymerisation) of microtubules (MT). The resulting perturbation of normal microtubule (MT) dynamics leads to the arrest of cell division in mitosis and, as a primary consequence, disrupts chromosomal movement (Ly and Cleveland, 2017; Čermák et al., 2020). Among such natural compounds, a particularly notable one is Taxol® (paclitaxel), this being the first reported microtubule-stabilizing agent (Yang and Horwitz, 2017). Docetaxel and cabazitaxel, which are taxane-based microtubule-stabilizing agents, are standard-of-care treatments for mCRPC and have demonstrated improved overall survival (Sousa-Pimenta et al., 2023; Hurwitz, 2015). However, clinical challenges persist including those related to drug resistance, dose-limiting toxicity and suboptimal progression-free survival. Such limitations have prompted the investigation of alternative microtubule-targeting agents with potentially distinct mechanisms of action and toxicity profiles (Yared and Tkaczuk, 2012). Despite ongoing efforts concerned with the discovery and development of new drugs, mCRPC remains a highly refractory disease.

**FIGURE 1:**
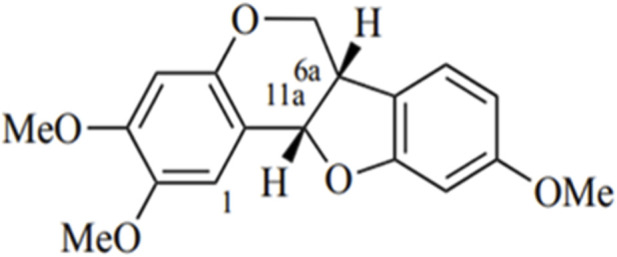
The structure of (+)-(6a*S*,11a*S*)-2,3,9-trimethoxypterocarpan with partial atom numbering.

Naturally-occurring pterocarpans are the second largest group of isoflavonoids and are characterized by the presence of a tetracyclic ring system comprised of mutually fused benzofuran and benzopyran subunits that embody two centers of chirality at the points of fusion, *viz*. at the C6a and C11a positions (see Figure 1) (Jimenez-Gonzalez et al., 2011; Selvam et al., 2017). Results from both *in vitro* and *in vivo* experiments support the concept that various pterocarpans are active against a range of cancers (Gaspar et al., 2021; Jimenez-Gonzalez et al., 2011; Rumjanek et al., 2019; Selvam et al., 2017). Within this large family of such compounds, our own focus has been a study of (+)-(6aS,11aS)-2,3,9-trimethoxypterocarpan [(+)-PTC, Figure 1], a natural product with significant potential as an anti-cancer agent. (+)-PTC was first isolated from *Platymiscium floribundum* and displayed notable cytotoxic effects in leukemia cell lines (Falcão et al., 2005; Militão et al., 2006; Militao et al., 2007) as well as against breast (Militao et al., 2014), ovarian (FARIAS et al., 2020) and prostate cancers (Moraes de Farias et al., 2022). Additionally, we have shown that in breast cancer cell lines (+)-PTC causes cell cycle arrest at prometaphase followed by impairment of bipolar spindle formation (Militao et al., 2014). *In silico* analyses based on molecular docking and molecular fragmentation studies, together with the use of conjugated caps or MFCC protocols, indicated that (+)-PTC has a low binding energy to tyrosine kinase (E.g.,5) (−219.09 kcal/mol). This suggested that the compound has the potential to inhibit Eg5 an important protein responsible for bipolar spindle formation (Farias et al., 2020; Hashimoto et al., 2020; Hashimoto et al., 2020). Previous studies from our group have demonstrated that (+)-PTC exhibits selective cytotoxicity against multiple cancer cell types including leukemia, breast, ovarian and prostate cancers Farias but minimal toxicity toward peripheral blood mononuclear cells (PBMCs; selectivity index >6-fold, IC_50_ >50 μM vs. 8.0 μM in PC-3 cells) (Moraes de Farias et al., 2022). Given this preliminary selectivity profile, an investigation of (+)-PTC’s mechanism of action in prostate cancer cells, as well as a comprehensive toxicity assessment in additional non-tumour cell types; is warranted. This is all the more so given that (+)-PTC also shows promising effects on the inhibition of bipolar spindle formation in the human prostate cancer cell line PC-3, this being an mCRPC cell line (Moraes de Farias et al., 2022). However, the molecular pathways and mechanisms by which (+)-PTC exerts such anti-cancer activity remain to be established. Accordingly, we sought to further investigate the anticancer potential of (+)-PTC, most particularly by studying the mechanism by which it promotes apoptosis and its impact on the global proteomic profile of PC-3 cells.

## 2 Materials and methods

### 2.1 Compound or chemical

A sample of (+)-(6a*S*,11a*S*)-2,3,9-trimethoxypterocarpan [(+)-PTC] (Figure 1) was synthesized as previously described (Farias et al., 2020). Its structure was confirmed by various means, most particularly by one- and two-dimensional NMR spectroscopic techniques, including COSY, HMQC and HMBC experiments as well as by an analysis of the associated physical properties and comparisons with relevant literature data.

### 2.2 Cell line and culture

The human prostate cancer cell line PC-3 used in the present studies was obtained from the National Cancer Institute, Bethesda, MD, USA. Cells were grown in RPMI-1640 medium supplemented with 10% fetal bovine serum, 2 mM glutamine, 100 μg/mL streptomycin and 100U/mL penicillin (type II, Sigma Chemical Co., St. Louis, Mo., USA) and incubated at 37 °C in a 5% CO_2_ and humidified atmosphere.

### 2.3 Apoptosis assay

PC-3 cells were plated on 24-well plates at a density of 1 × 10^5^ cells/mL and treated, for 24 h, with (+)-PTC or nocodazole at concentrations of 2 μM and 8 µM (in the case of the former compound) and of 0.25 µM (in the case of the latter compound). The negative control cells received 0.4% of DMSO. After treatment, the cells were transferred into microcentrifuge tubes and, while being maintained at room temperature, were centrifuged at 300 x g for 5 min. Harvested cells were then washed 3 times with ice-cold PBS and stained for 15 min with Annexin V-FITC (Thermorfisher™, Cat. No. V13246) in accordance with the manufacturer’s protocols. Thereafter, cells were stained for 5 min with DAPI (1 µM) (Merck™/Sigma-Aldrich, Cat. No. D9542). Stained cells were immediately analyzed by flow cytometry using a CytoFLEX flow cytometer (Beckman Coulter Life Sciences, Indianapolis, IN, USA) equipped with 405 nm (violet) and 488 nm (blue) lasers. For cell viability, DAPI fluorescence, involving excitation at 405 nm and detection through a 450/40 nm bandpass filter, was used. For Annexin V-FITC (an apoptosis marker), fluorescence involving excitation at 488 nm and detection through a 525/40 nm bandpass filter was employed. A minimum of 10,000 single events per sample were acquired at a flow rate of 30 μL/min. Forward scatter (FSC-Area vs. FSC-Height) was used to identify singlets and exclude doublets. Data were analyzed using FlowJo v10.10 software and applying the following gating strategy: (1) singlets were identified by FSC-A *vs*. FSC-H; (2) viable cells, early apoptotic cells, late apoptosis and necrosis were identified by V-450 (DAPI) *vs.* B-525(FITC). Differences between treatment groups and vehicle control were analyzed by one-way analysis of variance (ANOVA) followed by Dunnett’s multiple comparison test using GraphPad Prism 6.01 software (GraphPad Software, San Diego, CA, USA). Statistical significance was defined as p < 0.05.

### 2.4 Mitochondrial membrane potential (MMP)

MMP was assessed using Rhodamine 123 (Merck™/Sigma-Aldrich, Cat. No. R8004) and performed using minor modifications of the protocol described by Ferlini C and Scambia G (2007) [33]. Briefly, cells were plated on 24-well plates at a concentration of 1 × 105 cells per well. Cells were then incubated with (+)-PTC or nocodazole at concentrations of 2 μM and 8 µM and of 0.25 µM, respectively. Thereafter, cells were collected and centrifuged for 5 min at 300 x g. Harvested cells were washed once with FBS-free DMEM then incubated in 1 mL FBS-free DMEM with Rhodamine 123 (1ug/mL) at 37 °C for 30 min. Cells were then stained for 5 min with DAPI (1 µM) (Merck™, Sigma-Aldrich, Cat. No. D9542). Stained cells were immediately analyzed by flow cytometry using a CytoFLEX flow cytometer (Beckman Coulter Life Sciences, Indianapolis, IN, USA) equipped with 405 nm violet and 488 nm blue lasers. For cell viability, DAPI fluorescence, involving excitation at 405 nm and detection through a 450/40 nm bandpass filter, was used. For Rhodamine 123, fluorescence involving excitation at 488 nm and detection through a 525/40 nm bandpass filter was used. A minimum of 10,000 single events per sample were acquired at a flow rate of 30 μL/min. Forward scatter (FSC-Area vs. FSC-Height) was used to identify singlets and exclude doublets. Data were analyzed using FlowJo v10.10 software and involving the following gating strategy: (1) singlets identified by FSC-A vs. FSC-H; (2) V-405 (DAPI) to select live cells; (3) B-525 (Rhodamine 123) to analyze the MMP of live cells. The analysis was carried out using FlowJo software v10. Rhodamine 123 Mean Fluorescence Intensity (MFI) values were normalized relative to the vehicle control (DMSO 0.4%) and are expressed as a percentage of control. Differences between treatment groups and vehicle control were analyzed by one-way analysis of variance (ANOVA) followed by Dunnett’s multiple comparison test using GraphPad Prism 6.01 software (GraphPad Software, San Diego, CA, USA). Statistical significance was defined as p < 0.05.

### 2.5 Measurement of caspase activity

Caspase activity was detected using the Caspase-Glo 3/7 assay kit (Promega Corporation, Brazil). Briefly, PC-3 cells were seeded in 96-well white luminometer assay plates (Optiplate 96 well white, cat 6055290, Revvity) at a density of 5 × 10^4^ cells per well and incubated at 37 °C. Cells were treated with vehicle, (+)-PTC or nocodazole for 24 h. 100 μL caspase 3/7 reagents were added to each well and incubated for 1 h at room temperature. The luminescence intensity was measured using a SpectraMax iD3 multi-mode microplate reader (Molecular Devices, LLC, San Jose, CA).

### 2.6 Western blot analysis

The proteins (20 μg) were subjected to 12% gel electrophoresis and transferred onto PVDF Transfer membrane (cat 88518, Thermofisher). The membranes were incubated for 1 h at room temperature in blocking buffer (5% skim milk in TBS-T) and then incubated with the following antibodies; anti-caspase-7 antibody (dilution 1:500) (cat 9492, Cell Signalling Technology, USA); anti-Bax antibody (cat 5023S, Cell Signalling Technology, USA) overnight at 4 °C. After washing with TBS-T, the membranes were incubated with horseradishperoxidase-conjugated anti-rabbit antibody (1:2,000); (cat 7074P2, Cell Signalling Technology, USA) or anti-GAPDH (cat MA5-15738-HRP, Thermofisher) for 2 h at room temperature. Detection was performed using Pierce ECL Western (cat 32106, Invitrogen, Thermofisher) and imaged using Ibright 1500 (Invitrogen, Thermofisher). Data are from three independent experiments.

### 2.7 Global proteomic assay

#### 2.7.1 Sample preparation and protein extraction

PC-3 cells were plated on 6-well plates at a concentration of 1 × 10^6^ cells per well and were treated with 8.0 µM (+)-PTC for 24 h. The same incubation time was used for the negative control (*viz.* 0.4% DMSO) and the two positive controls, namely, monastrol (50 µM) and nocodazole (0.25 µM). All treatments were performed in triplicate and two biological replicates and the samples pooled to reduce biological variation. The cells were harvested with trypsin for 10 min and lysates were transferred into 15 mL conical tubes. Lysis buffer (comprising 8 M urea, 75 mM NaCl, 50 mM Tris, pH 8.2) was added to each tube and one tablet of protease cocktail inhibitor (ex. Roche Diagnostics, Indianapolis, IN, USA) then added per 25 mL of buffer (Villén and Gygi, 2024). The cells were sonicated (60 s, 4 °C) three times at 2 min intervals using a sonicator operating at an output of 15% of maximum amplitude. Thereafter, the cells were centrifuged at 2,500 x g and 4 °C for 10 min so as to eliminate cellular debris and the supernatant then transferred into new tubes. Equal amounts of total protein (40 μg) were used for LC-MS/MS analysis as determined by (ThermoFisher Scientific) Qubit® quantification.

#### 2.7.2 LC/MSE quantification studies

Quality control samples of PC-3 cells were prepared by taking one randomly selected sample from the negative control group and this then being divided into multiple aliquots. These were added to each sample preparation batch then processed and analyzed in parallel with the normal samples. Buffer exchange into 50 mM ammonium bicarbonate was carried out using spin columns (Millipore, USA) with a 5 kDa molecular cut-off. Disulfide bonds associated with the proteins were reduced using 100 mM dithiothreitol (Sigma, UK) at 60 °C for 0.5 h and the ensuing free sulfhydryl moieties were alkylated with 200 mM iodoacetamide (Sigma) in the dark at room temperature for 0.5 h. The proteins were then enzymatically digested using porcine trypsin (Promega, USA) at a ratio of 1:50 (w/w trypsin/protein) for 17 h at 37 °C. Reactions were quenched through the addition of 8.8 M HCl (1:60 v/v ratio) to each sample that were then were stored at −80 °C and the extracted peptides were re-suspended in 0.1% aqueous formic acid (FA) solution. LC-MSE profiling was carried out in expression mode using an ACQUITY UPLC M-Class SYNAPT G2-Si UPLC/MS/MS system (Waters, UK) as described previously (Levin et al., 2010). The ensuing data were processed using the ProteinLynx Global Server (PLGS) v.2.4 (Waters). Qualitative and quantitative proteomic analyses were performed in a bidimensional nano-UPLC tandem nano-ESI-MSE platform by multiplexed data-independent acquisition (DIA) experiments. The peptides (1 μg) were injected into a 2D-RP/RP Acquity UPLC M-Class System (Waters Corporation, Milford, MA) interfaced with a Synapt G2-Si mass spectrometer (Waters Corporation, Milford, MA). The samples were fractionated in first-dimension chromatography with an XBridge Peptide BEH C18 NanoEase Column (130 Å, 3.5 μm, 300 μm × 50 mm, Waters Corporation, Milford, MA). Peptide elution was performed using discontinuous steps of acetonitrile (11%, 14%, 17%, 20% and 50% acetonitrile) for 10 min at a flow rate of 2,000 nL/min. After each step, peptide loads were carried to a second-dimension separation using an ACQUITY UPLC HSS T3 nanoACQUITY Column (100 Å, 1.8 μm, 75 μm × 150 mm, Waters Corporation, Milford, MA). Peptide elution was achieved using an acetonitrile gradient from 7% to 40% (v/v) for 54 min at a flow rate of 500 nL/min directly into a Synapt G2-Si.

The mass spectrometer was operated in resolution mode with a *m/z* resolving power of about 35,000 FWHM using ion mobility with a cross-section resolving power of at least 40Ω/ΔΩ. The effective resolution obtained with the conjoined ion mobility was 1 800 000 FWHM. MS/MS analyses were performed by nano-electrospray ionization in positive ion mode nano-ESI (+) and using a NanoLock Spray (Waters, Manchester, UK) ionization source. The lock mass channel was sampled every 30 s. The mass spectrometer was calibrated with an MS/MS spectrum of [Glu1]-Fibrinopeptide B human (Glu-Fib) solution and was delivered through the reference sprayer of the using NanoLock Spray source.

### 2.8 Data base search and proteome quantification

Progenesis QI V2.0 (QIfp) for proteomics (Nonlinear Dynamics/Waters, UK) was used for automated data processing and data-base searching. The generated peptide masses were searched against the UniProt species-specific protein sequence data-base using the Progenesis QI V2.0 (QIfp) for proteomics for protein identification and quantification (Nonlinear Dynamics/Waters, UK). Feature extraction, chromatographic/spectral alignment, data filtering and statistical analyses were performed. All runs were selected for detection with an automatic detection limit. Features within room temperature (RT) ranges of 0–5 min and 45–65 min were filtered out as were features with charge ≥ +8. A normalization factor was then calculated for each run to account for differences in sample loadings between injections. The experimental design was set up to group multiple injections from each run. An algorithm was then used to calculate and tabulate raw and normalized abundances, max fold change and ANOVA p values for each feature in the data set. The features were tagged in sets based on characteristics such as MS/MS > 1 and p < 0.01. The initial ion-matching requirements were ≧2 fragments per peptide, ≧5 fragments per protein and ≧1 peptide per protein. The enzyme specificity was defined by trypsin and one missed cleavage was allowed. For database searching the search parameters included: Variable modifications-Carbamidomethyl (Cys, fixed), Oxidation (Met, variable); peptide mass tolerance - ±20ppm; fragment mass tolerance -±0.4Da; and with Decoy searching used to define the false discovery rate (FDR). The significance threshold of the ion score was calculated based on a false discovery rate of ≤1%. The data were filtered to show only statistically significant differences (p < 0.05, ANOVA) coupled with a change in protein abundance of 1.5-fold or greater. Additionally, absolute quantification was performed using ADH as an internal standard so as to give an absolute amount of each identified protein/peptide.

The mass spectrometry proteomics data have been deposited to the ProteomeXchange Consortium via the PRIDE (Perez-Riverol et al., 2025) partner repository with the dataset identifier PXD073655 and 10.6019/PXD073655.

### 2.9 Bioinformatic analyses

Log Fold Changes (logFCs) were calculated to identify differentially expressed proteins (DEPs) across all treatments [*viz.* those using nocodazole, (+)-PTC and monastrol] compared to the negative control. Genes with a fold change below 1.5, duplicated entries and those with an “infinity” Max Fold Change were excluded. A heatmap was generated using the *pheatmap* package in R to visualize and compare upregulated and downregulated proteins across the three treatments.

All DEPs identified through LC/MSE were initially categorized according to their corresponding PANTHER protein classes (Mi and Thomas, 2009). Next, statistical enrichment analyses for biological processes, molecular functions and cellular components of the DEPs were carried out using the Gene Ontology (GO) consortium data-base by inputting the DEP list as query terms into STRING (Search Tool for the Retrieval of Interacting Genes/Proteins) (Szklarczyk et al., 2023). By applying a right-sided hypergeometric multiple correction test using the Benjamini–Hochberg method, the GO terms with a p-value < 0.01 were considered statistically significant. Protein-protein interaction (PPI) networks for all DEPs were then generated in STRING, utilizing the *Homo sapiens* data-base with a confidence score of 0.40. DEPs were mapped to Reactome pathways (Szklarczyk et al., 2023) and an over-representation test was conducted with a false discovery rate (FDR) cut-off of < 0.01 so as to select for the enriched pathways.

## 3 Results

Prior to conducting the proteomics assays; the mechanism of cell death in PC-3 cells treated with (+)-PTC was evaluated. Thereafter, the expressed proteins in the (+)-PTC-treated cells were compared with those associated with the monastrol- and nocodazole-treated cells as well as the negative control in the global proteomic assay.

### 3.1 Apoptosis assay of (+)-PTC- and nocodazole-treated PC-3 cells

**FIGURE 2:**
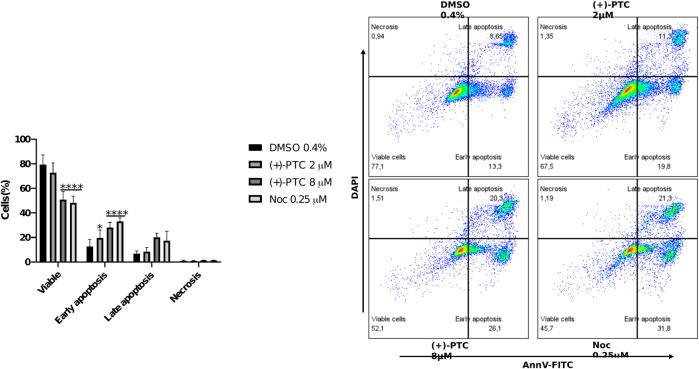
(+)-PTC induces concentration-dependent apoptosis in PC-3 cells. **(A)** % of viable, early apoptotic, late apoptosis and necrosis of PC-3 cells across different treatments groups. **(B)** Representative Annexin V-FITC/DAPI flow cytometry dot plots. Quadrants: lower left, viable (Annexin V−/DAPI−); lower right, early apoptotic (Annexin V+/DAPI−) indicating genuine apoptosis; upper right, secondary necrotic (Annexin V+/DAPI+). (+)-PTC induces dose-dependent shift toward the early apoptotic quadrant. Ten thousand events were acquired in each experiment. Data are derived from three independent experiment performed in triplicate. *p < 0.05; ****p < 0.001.

Cell viability and apoptosis induction were assessed in PC-3 cells following 24 h treatment with (+)-PTC or nocodazole using Annexin V-FITC/DAPI (Figure 2). As so revealed, (+)-PTC did induce concentration-dependent apoptosis and loss of cell viability. At 2 μM, (+)-PTC did not significantly decrease overall cell viability yet induced early apoptosis (19.8% early apoptotic cells; p < 0.05) (Figure 2). At 8 μM, (+)-PTC produced marked loss of cell viability (52.1% viable cells; p < 0.001) and this was accompanied by elevated early apoptotic populations (26.1%; p < 0.001) (Figure 2). Similarly, nocodazole (0.25 µM) reduced viable cell populations to 45.7% (p < 0.001) with 31.8% early apoptotic cells (p < 0.001), this being comparable to the high-dose (+)-PTC treatment.

### 3.2 Mitochondrial membrane potential (MMP) of (+)-PTC and nocodazole-treated PC-3 cells

**FIGURE 3:**
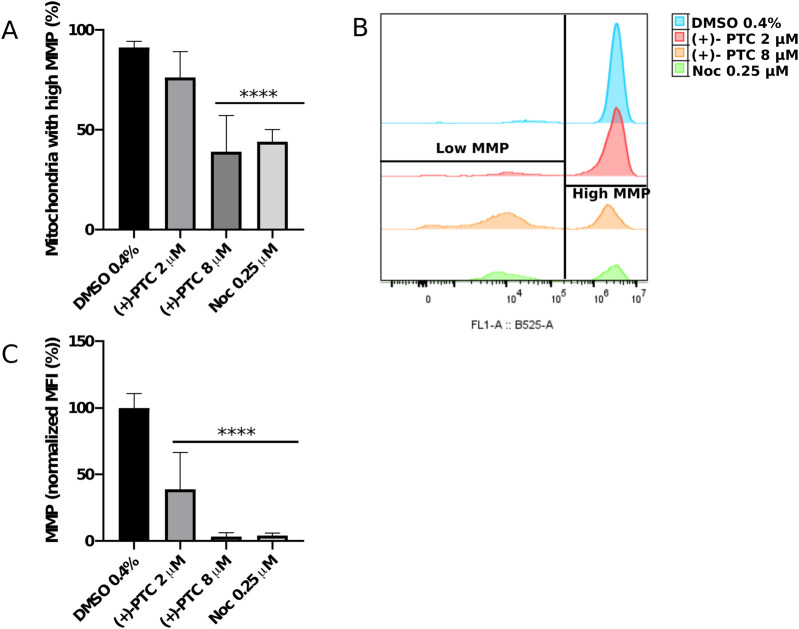
(+)-PTC induces concentration-dependent loss of a mitochondrial membrane potential in PC-3 cells. **(A)** % of mitochondria from live cells with high MMP. **(B)** Representative flow cytometry histograms or dot plots showing rhodamine 123 fluorescence (a mitochondrial membrane potential indicator) in PC-3 cells treated with vehicle control (DMSO 0.4%), (+)-PTC (2 or 8 µM), or nocodazole (0.25 µM) for 24 h. Gates delineate populations with high MMP (rhodamine 123-positive-shift to the right) and low MMP (rhodamine 123-negative/low-shift to the left) cells. **(C)** Quantification of rhodamine 123 mean fluorescence intensity (MFI) (a mitochondrial membrane potential indicator) normalized to vehicle control (DMSO 0.4) across treatment groups. Ten thousand events were acquired in each experiment. Data are derived from three independent experiments performed in triplicate. *p < 0.05; ****p < 0.001.

Following 24 h treatment, the mitochondrial membrane potential (MMP) in PC-3 cells was analyzed using rhodamine 123 (Figure 3). This revealed that (+)-PTC-induced, concentration-dependent depolarization of mitochondrial membranes. At 2 μM, (+)-PTC significantly decreased rhodamine 123 MFI to 38.9% ± 10.9% compared to negative control (p < 0.001) and without significant reduction of high MMP cells (Figures 3A–C). At 8 μM, (+)-PTC markedly reduced rhodamine 123 MFI to 3.3% ± 1.1% of control (p < 0.0001) and substantially decreased high MMP cells to 39.08% ± 18.04% (p < 0.0001) (Figures 3A–C). Nocodazole (0.25 µM) induced comparable depolarization, reducing MFI to 4.1% ± 0.8% of control (p < 0.0001) with high MMP cells declining to 44.15% ± 6.00% (Figures 3A–C).

**FIGURE 4:**
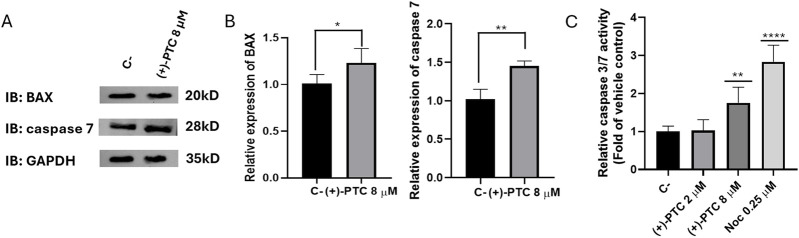
Treatment with (+)-PTC increases the expression of apoptotic proteins after 24 h **(A, B)** Western blot analysis of Bax and caspase-7 expression in PC-3 cells with DMSO 0.4%, (+)-PTC 8 µM. **(C)** Caspase-Glo 3/7 assay analysis showing (+)-PTC-induced caspase 3/7 activity in PC-3 cells. The data are presented as the mean ± SD. (**) p < 0.001; (*) p < 0.05.

Western blot analyses and caspase-3/7 activity assays were performed to assess the activation of the intrinsic apoptotic pathway in PC-3 cells treated with (+)-PTC for 24 h. The results revealed a significant increase in the expression of caspase-7 and Bax levels after treatment with (+)-PTC compared to the negative control (p < 0.05) (Figures 4 A,B). Additionally, caspase-3/7 enzymatic activity was significantly elevated at 8 µM (+)-PTC compared to the negative control (p < 0.05), as measured by luminescence assay (Figure 4C).

### 3.3 Proteomic analysis of (+)-PTC-, monastrol- and nocodazole-treated PC-3 cells

**FIGURE 5:**
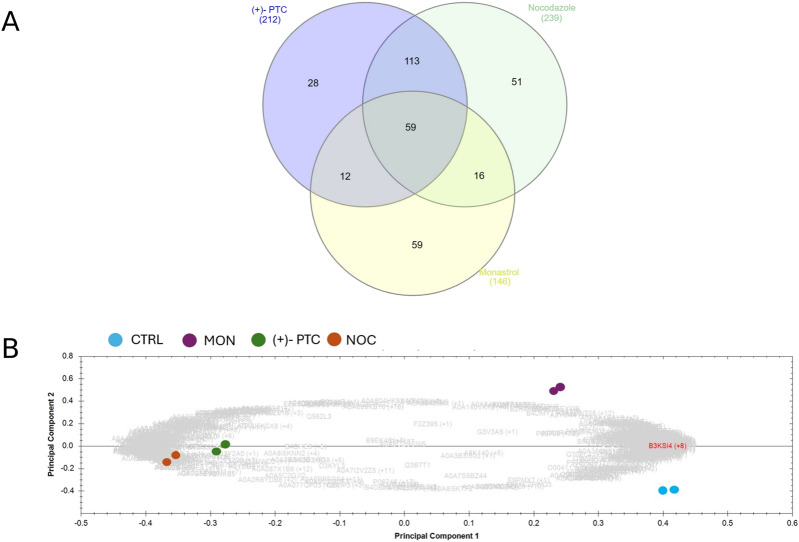
Evaluation of differentially expressed proteins (DEPs) in PC-3 cells after different treatments. **(A)** Venn diagram showing the distribution of DEPs across the three different treatments [involving (+)-PTC, nocodazole]. Unique proteins are represented in non-overlapping areas, while shared proteins are displayed in the overlapping sections. The numbers indicate the total proteins in each category or intersection; **(B)** Principal Component Analysis (PCA) showing the similar proteomic profiles of the PC-3 cells after treatment with (+)-PTC or nocodazole. Only ANOVA-significant proteins (q-value < 0.05) were chosen across the sampling treatments when compared to negative control (CTRL). CTRL: Negative control; NOC: nocodazole; MON: monastrol.

**FIGURE 6:**
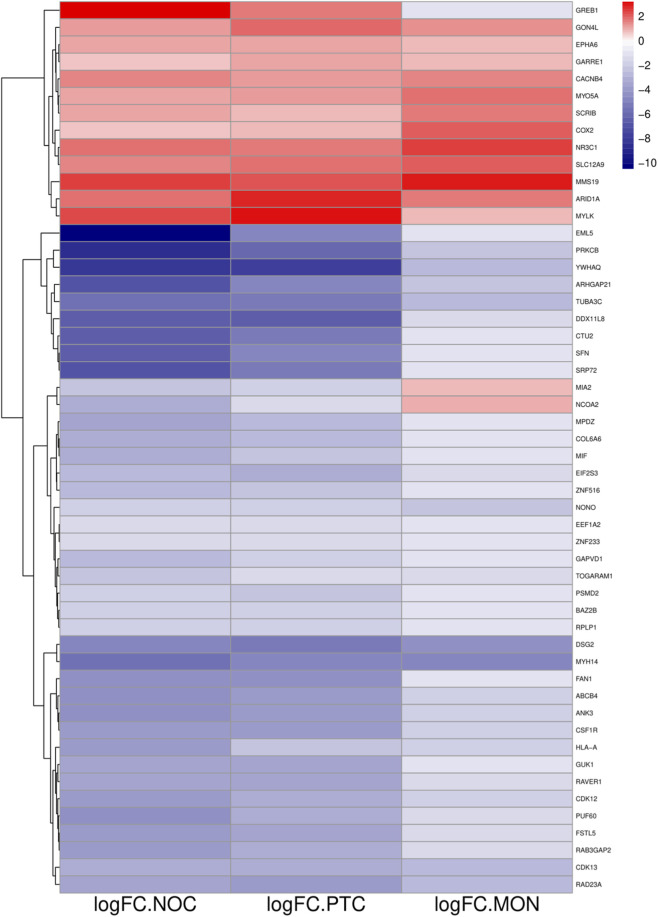
Differentially Expressed Protein (DEP) comparisons between PC-3 cells treated with (+)-PTC, nocodazole and monastrol. Scale colors: Red (Upregulated); Blue (Downregulated) CTRL: Negative control; NOC: nocodazole; PTC: (+)-PTC; MON: monastrol.

After considering a false discovery rate of 1% and at least one unique peptide to ensure redundancy control, there were 952 proteins identified as a result of the (+)-PTC treatment of PC-3 cells. As compared to the negative control, PC-3 cells treated with (+)-PTC, nocodazole and monastrol had 212 (Supplementary Table S1), 239 (Supplementary Table S2) and 146 (Supplementary Table S3) DEPs, respectively. More particularly, (+)-PTC treatment induced the upregulation of 68 proteins and the downregulation of 144. Similarly, nocodazole upregulated 65 proteins and downregulated 174, while monastrol upregulated 61 proteins and downregulated 85 in comparison with the negative control.

(+)-PTC-treated cells showed 172 proteins in common with those exposed to nocodazole but just 71 in common with the monastrol-treated cells (Figure 5A). This analysis indicates that the proteomes of (+)-PTC- and nocodazole-treated cells show close clustering. The proteome pattern of (+)-PTC-treated cells appeared similar to the analogous pattern of nocodazole-treated cells as revealed by principal component analyses (PCA) (Figure 5B). On the other hand, there were fewer similarities among the proteomes of those cells treated with (+)-PTC, monastrol and the negative control (Figure 5B). Moreover, heatmap analysis of the 59 DEPs associated with the cells derived from the (+)-PTC- and positive control-treatments highlights the similarities between the effects of (+)-PTC and nocodazole treatments rather than between the (+)-PTC and monastrol treatments (Figure 6).

The distributions of the 172 differentially expressed proteins arising from (+)-PTC and nocodazole treatments of PC-3 cells are presented in Supplementary Figure S1. The five proteins with the greatest abundance in (+)-PTC-treated cells (as compared to those resulting from the nocodazole treatment regime) are those arising from the TTLL3, ANAPC7, PIK3CA, ARID4B and COL16A1 protein-encoding genes. In terms of the downregulated protein distributions, the top five for (+)-PTC-treated PC-3 cells were those associated with the KDM2B, PTOV1, YWHAQ, DDX11L8 and PRKCB genes while the ones arising from the nocodazole treatment were those derived from the corresponding EML5, KDM2B, PRKCB, YWHAQ and TABG3 genes (Supplementary Table S4).

### 3.4 Gene ontology enrichment and protein interaction

**FIGURE 7:**
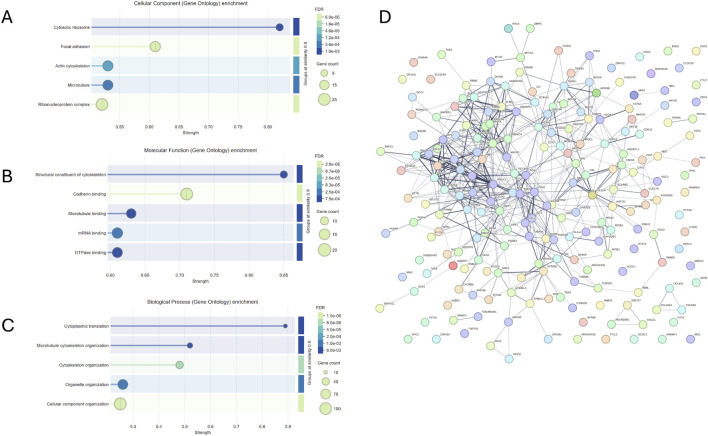
Proteomic profile and gene ontology enriched terms for the DEPs arising from treatment of the PC-3 cells with (+)-PTC. **(A)** GO functional enrichment analysis for biological processes associated with DEPs; **(B)** GO functional enrichment analysis for molecular functions associated with DEPs; **(C)** GO functional enrichment analysis for cellular components associated with DEPs; **(D)** Protein-protein interaction networks associated with DEPs.

To understand the biological significance of the DEPs found in the (+)-PTC- and nocodazole-treated PC-3 cells, their functional classifications were assessed using protein enrichment Gene Ontology (GO) analysis and involving the display of all up- and downregulated proteins for each treatment. Such analyses were performed using the association criteria of FDR p < 0.01 and the strength metric. Amongst the DEPs observed in the (+)-PTC-treated cells, the GO biological processes with the highest enrichment were “cytoplasmic translation”, “microtubule cytoskeleton organization”, “cytoskeleton organization”, “organelle organization” and “cellular component organization” (Figure 7A).

In terms of molecular function, the most enriched GO terms were “structural constituent of cytoskeleton”, “cadherin binding”, “microtubule binding”, “mRNA binding” and “GTPase binding” (Figure 7B) while for cellular components the top five enriched terms were “cytosolic ribosome”, “focal adhesion”, “actin cytoskeleton”, “microtubule” and “ribonucleoprotein complex” (Figure 7C). The enrichment analysis for nocodazole-treated PC-3 cells was similar to that encountered after the (+)-PTC treatment regime (Supplementary Figure S2). The overlapping or shareable terms between both treatments were related to “structural constituents of cytoskeleton” and “cadherin binding”. The protein-protein interaction (PPI) network of DEPs in the (+)-PTC-treated PC-3 cells generated 207 nodes and 508 edges with a PPI enrichment p-value of 1.0^−16^ (Figure 7D).

### 3.5 Protein identification and classification

Proteomic analyses of the DEPs encountered in the negative control group [relative to those in the (+)-PTC-treated PC-3 cells] revealed a set of proteins exclusively expressed in this group. As revealed through analysis using the PHANTER knowledge base, these DEPs belonged to four different classes, *viz.* microtubule binding motor proteins, actin-binding motor proteins, DNA ligases and RNA metabolism (Table 1).

**TABLE 1:** List of proteins exclusively regulated by (+)-PTC treatment in comparison to the negative control.

Proteins exclusively expressed in the negative control		
Gene name	Protein class	Protein description
COMMD8*	----	COMM domain-containing protein 8
DNAH14	Microtubule binding motor protein	Dynein axonemal heavy chain 14
NUP85	Structural protein	Nuclear pore complex protein Nup85
TRIP11	Actin-binding motor protein	Thyroid receptor-interacting protein 11
ALAS1	Transaminase	5-Aminolevulinate synthase, non-specific, mitochondrial
TKT	Transketolase	Transketolase
FBXW7	Ubiquitin-protein ligase	F-box_WD repeat-containing protein 7
CCDC39*	----	Coiled-coil domain-containing protein 39
RABEPK*	----	Rab9 effector protein with kelch motifs
POGLUT3*	----	Protein O-glucosyltransferase 3
LIG1	DNA ligase	Leucine-rich repeats and immunoglobulin-like domains protein 1
NRXN2	Cell adhesion molecule	Neurexin-2
EIF4G1	Translation initiation factor	Eukaryotic translation initiation factor 4 gamma 1
G3BP1	RNA metabolism protein	Ras GTPase-activating protein-binding protein 1
CHD3	Chromatin/chromatin-binding, or -regulatory protein	Chromodomain-helicase-DNA-binding protein 3
HLA-A	Major histocompatibility complex protein	HLA class I histocompatibility antigen, A alpha chain
Proteins exclusively expressed in the (+)-PTC-treated group		
ITGB5	Integrin	Integrin beta-5
SMTN	Scaffold/adaptor protein	Smoothelin
WDR17*	----	WD repeat-containing protein 17

* These proteins do not have a specific protein class in the PANTHER, database.

**FIGURE 8:**
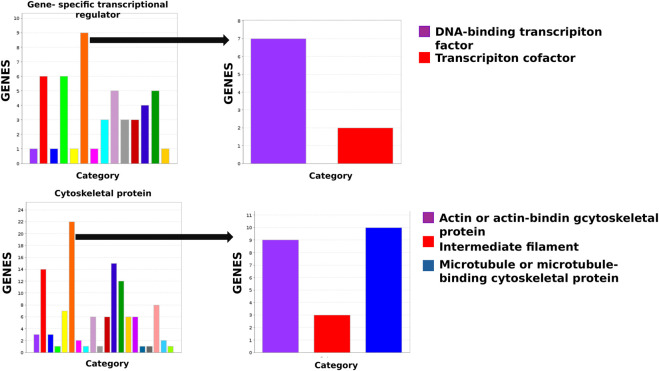
Protein classes of the DEPs observed in PC-3 cells treated with (+)-PTC. **(A)** Upregulated proteins in the (+)-PTC-treated PC-3 cells fall, in the main, into the “gene specific transcriptional regulator” class which is comprised of two categories; **(B)** Downregulated proteins in the (+)-PTC-treated PC-3 cells fall, in the main, into the “cytoskeletal protein” class which is comprised of three categories. The black arrow indicates the different subcategories that belong to the most relevant class for upregulated and downregulated proteins.

The DEPs observed in the (+)-PTC-treated PC-3 cells were assigned to their relevant protein classes by mapping them using the PANTHER system. The upregulated proteins were grouped into 12 classes (Supplementary Table S1), the five most significant being the “gene-specific transcriptional regulator”, “protein modifying enzyme”, “transporter”, “cytoskeletal proteins and “transmembrane signal receptor” ones. As stated above, most of the upregulated proteins were classified as gene-specific transcriptional regulators. In addition, this protein class was divided into two sub-categories (Figure 8A), *viz.* the DNA-binding transcription factor and transcription cofactor groups and so revealing that the former is the more significant, this being composed of five DEPs including those associated with the ligand-dependent nuclear receptor corepressor-like protein (LCORL), glucocorticoid receptor (NR3C1) and transcription factor 12 (TCF12) genes (Supplementary Table S5).

The downregulated proteins were assigned to 20 classes, the five most relevant being “cytoskeletal protein”, “RNA metabolism protein”, “metabolite interconversion enzyme”, “protein modifying enzyme” and “translational protein” groups (Figure 8B). Moreover, the main protein class, *viz*. the “cytoskeletal protein” one, was divided into three sub-categories, namely, the “actin or actin-binding cytoskeletal protein”, “microtubule or microtubule-binding cytoskeletal protein” and “intermediate filament” ones. The actin or actin-binding cytoskeletal protein class is represented by eight protein-encoding genes including CFL1, POTEF, PFN1, ACTN4 and FAN1 (Supplementary Table S6). Within the microtubule-binding cytoskeletal protein category, a total of eight proteins were identified including ones associated with the EML5, TUBA4A, MAP1B, TUBA1B and KIF5A genes (Supplementary Table S6).

### 3.6 Pathway analysis

An analysis, using the negative control as reference, of all of both the up- and downregulated DEPs in the (+)-PTC-treated PC-3 cells, through mapping into the Reactome data-base, allowed for the identification of pathways modulated by the compound to be identified. An over-representation analysis was performed with a p-value ≤ 0.01 and the five most relevant pathways thereby selected (Table 2). This revealed that “nuclear receptor transcription pathway”, “RAC1 GTPases cycle”, “RHO GTPase cycle”, “NEP/NS2 interactors with the cellular export machinery” and “RHOB GTPase effectors” are involved in the (+)-PTC-mediated impacts on PC-3 cells (Table 2). Specifically, the “microtubule-dependent trafficking of connexons from Golgi to the plasma membrane”, “transport of connexons to the plasma membrane”, “carboxyterminal post-translational modifications of tubulin”, “L13a-mediated translational silencing of ceruloplasmin expression” and “RHO GTPases activate IQGAPs” were the pathways associated with the downregulated proteins arising from the (+)-PTC treatment regime (Table 2). The proteins regulated through the nocodazole treatment regime revealed the operation of similar pathways (Supplementary Table S7).

**TABLE 2:** The top five most significant enriched Reactome pathways associated with DEPs identified in (+)-PTC-treated PC-3 cells (relative to the negative control group).

Pathway name	p-value
Upregulated protein	
Nuclear receptor transcription pathway	2.48^–8^
RAC1 GTPases cycle	5.5^–4^
RHO GTPase cycle	9.52^–4^
NEP/NS2 interactors with the cellular export machinery	6.16^–3^
RHOB GTPase cycle	7.4^–3^
Downregulated protein	
Microtubule-dependent trafficking of connexons from golgi to the plasma membrane	3.09^–6^
Transport of connexons to the plasma membrane	3.83^–6^
SRP-dependent cotranslational protein targeting to membrane	2.74^–5^
L13a-mediated translational silencing of ceruloplasmin expression	2.84^–5^
Carboxyterminal post-translational modifications of tubulin	7.4^–3^

Among the enriched Reactome pathways associated with the upregulated proteins, three are strongly linked to the GTPase cycle. The protein coding genes contributing to these pathways and found to be upregulated in (+)-PTC-treated PC-3 cells include DOCK2, GARRE1, PIK3CA, ECT2, CIT, SPTAN1, PHIP, POTEE, and SCRIB. In contrast, the predominant Reactome pathways associated with the downregulated proteins are mostly involved in transport and trafficking to plasma membranes. The protein-coding genes implicated in these pathways and significantly downregulated in the treated cells are TUBA4A, TUBA3C, TUBA1B, and TUBB8B.

## 4 Discussion

Previously, a report evaluating the antiproliferative effects of (+)-PTC on PC-3 and LNCaP cell lines demonstrated time-dependent cytotoxicity with IC_50_ values of 8.0 µM and 5.6 µM respectively, while exhibiting minimal toxicity toward peripheral blood mononuclear cells (PBMCs) (IC_50_ > 50 µM) and so indicating selectivity for cancer cells over normal immune cells (Moraes de Farias et al., 2022). These findings prompted further investigation into the mechanism underlying the (+)-PTC-induced growth inhibition. Subsequent cell cycle analysis revealed a marked accumulation of PC-3 cells in G2/M phase following 24 h treatment with (+)-PTC (Supplementary Figure S3) (Moraes de Farias et al., 2022), suggesting interference with mitotic progression. Immunofluorescence analysis with α-, β-, and γ-tubulin antibodies demonstrated that (+)-PTC disrupts normal bipolar spindle formation, leading to monopolar and multipolar spindles in >80% of mitotic PC-3 cells and this was accompanied by disrupted γ-tubulin distribution and disorganized centrosomes (Supplementary Figure S4; Supplementary Figure S5) (Moraes de Farias et al., 2022). Such impacts on bipolar spindle formation can provide an important means for treating cancer because different forms of these undergo mutations and genetic alterations related to the mitotic phase (Van Vuuren et al., 2015).

Given that prolonged mitotic arrest and impairment of bipolar spindle formation is a trigger for the induction of apoptosis (Ruan et al., 2018) we investigated whether (+)-PTC-mediated cell cycle arrest leads to apoptotic cell death. In our study, it was observed that such treatment, as occurred after 24 h, promoted the externalization of phosphatidylserine (PS), which occurs during early apoptosis and serves as a recognition point to remove apoptotic cells (Zhou, 2007). To shed light on the possible pathway to apoptosis activated by (+)-PTC, we investigated the MMP of PC-3 cells and the proteins involved in the apoptosis pathway (BAX and Caspase-7). Our results showed elevated BAX levels, loss of mitochondrial membrane potential and caspase activation, these being consistent with engagement of the intrinsic apoptotic pathway. Apoptosis is a programmed cell death regulated through different mechanisms that involves two different pathways (extrinsic and intrinsic), the intrinsic one being dependent on mitochondria. The intrinsic pathway is activated by intracellular stress, such as microtubule disruption, ROS and DNA damage, leading to mitochondrial outer membrane permeabilization (MOMP) due to of activation BAX/BAK that promotes formation pore in the mitochondria with subsequent release of cytochrome C and activation of effector caspase 3 and 7 (Qi et al., 2025; Rovini et al., 2011). The loss of MMP is an important event in intrinsic apoptosis, preceding caspase three and seven activation and irreversible cell death commitment (Kroemer et al., 2007). In the context of cancer therapy, intrinsic pathway activation offers certain mechanistic advantages. First, MOMP represents a largely irreversible event that commits cells to death even in the presence of downstream caspase inhibition (Lopez and Tait, 2015). Second, cancer cells can evade extrinsic pathway-mediated apoptosis through overexpression of cellular FLICE-inhibitory protein (c-FLIP) or downregulation of death receptors (Safa and Pollok, 2011; Ivanisenko and Lavrik, 2022).

The temporal relationship observed in our study, wherein PS externalization and cell viability loss closely parallel mitochondrial depolarization, increased BAX and caspase-7 activation indicate a possible role of the intrinsic pathway as the primary mechanism of (+)-PTC-induced apoptosis in PC-3 prostate cancer cells. These findings are consistent with our previous time-course analysis showing that prolonged G2/M arrest (24 h) is followed by mitotic catastrophe characterized by multinucleated cells and apoptotic features at 48–72 h (Supplementary Figure S6) (Moraes de Farias et al., 2022). This suggests that the apoptotic signaling detected at 24 h in the current study may represent early commitment to cell death that manifests fully after prolonged mitotic arrest.

The current proteomic study was designed to provide molecular-level insights into how (+)-PTC causes cell cycle arrest in mitosis and which proteins are modulated that lead to the functional disruption of bipolar spindles, something we had already demonstrated (Supplementary Figures S3-5) (Moraes de Farias., et al., 2020; Moraes de Farias et al., 2022). To probe the anti-mitotic effects previously displayed by (+)-PTC this was compared with nocodazole (an MDA) and monastrol (an Eg5 inhibitor).

Proteomic analyses showed that PC-3 cells treated with (+)-PTC and nocodazole generated similar DEP profiles (Figures 5, 6). Relative to the negative control, the tubulin protein coding gene TTLL3 was one of the five most upregulated observed in both the (+)-PTC- and nocodazole-treated cells. This gene promotes post-translational modifications by adding glycine residues to tubulin and so positively affecting the proper assembly and function of microtubules (Tian and Qin, 2019; Wloga et al., 2009). In the context of cancer, it is known that the downregulation of tubulin tyrosine ligase proteins is associated with tumour growth and genomic instability. Moreover, downregulation of TTLL3, VASH2 and HDAC6 is implicated in the metastatic process and cell proliferation in prostate cancer (Lee et al., 2008; Lopes and Maiato, 2020). Additionally, the protein kinase gene PRKCB is one of the most prominently downregulated observed in (+)-PTC- and nocodazole-treated cells. PRKCB belongs to the protein kinase family that act by phosphorylating a range of proteins related to cell proliferation, cell cycle and migration (Abdelatty et al., 2022; Poole et al., 2004). PRKCB is over-expressed in prostate cancer, and it has been linked to the promotion of a favorable tumour micro-environment for cancer cell proliferation in mouse mammary tumors (He et al., 2022; Wallace et al., 2014; Zamora et al., 2023). For example, the compound enzastaurin reduced, through PRKCB inhibition, neuroendocrine features and enhanced the *in vivo* effect of docetaxel and so demonstrating that reduced expression of this kinase contributes positively to tumor regression (Blanc et al., 2023). Accordingly, we surmise that the alterations of TTLL3 and PRKCB expression induced by (+)-PTC contributes, in association with other factors, to its anti-cancer activity in prostate cancer cells.

Relative to the negative control, the gene ontology functional analysis detailed above reveals a relevant involvement of DEPs in (+)-PTC-treated PC-3 cells arising through the drug’s impact on cytoplasmic translation as well as on microtubule and cytoskeletal organization (Figure 6A). That group of proteins correlated to cytoplasmic translation was significantly downregulated in (+)-PTC-treated PC3 cells with these consisting, primarily, of eukaryotic translation initiation factors (EIF) and ribosomal proteins. In response to both extrinsic and intrinsic stress stimuli, cells activate the integrated stress response (ISR) system that focuses on reducing protein synthesis through phosphorylation of EIF-related proteins so as to prevent cell death. However, under severe stress, this can activate cell death pathways (Pakos-Zebrucka et al., 2016; Tian et al., 2021). Vicente and collaborators (2024) (Vicente et al., 2024) evaluated two microtubule targeting agents (MTAs) in different cancer cell lines associated with solid tumors and after 24 h of treatment it was shown that nocodazole increased the number of cells in mitosis, activated the ISR system and decreased protein synthesis. This was followed by alterations in EIF protein expression. Taken together, our results correlate with these findings, as most DEPs were downregulated after (+)-PTC treatment. Additionally, (+)-PTC altered the expression of proteins related to the ISR system and ribosomes.

Relative to the negative control, (+)-PTC-treatment promoted both microtubule and cytoskeletal organization, these being related to the regulation of cellular processes dependent on cytoskeletal proteins. Analysis of the downregulated proteins reveals that a significant proportion of them belong to the cytoskeletal protein class and so highlighting the potential impact of the treatment on cell integrity and growth dynamics (Figure 6). Notably, most of these proteins are tubulin or tubulin-related ones and were found to be downregulated in (+)-PTC-treated PC-3 cells. Tubulin is the main component of microtubules which, along with actin and intermediate filaments, comprise the cellular cytoskeleton. Microtubules play a crucial role in various cellular processes, including structural organization, intracellular transport, cell polarity and mitotic spindle formation (Goodson and Jonasson, 2018; Mcintosh, 2016). The observed downregulation of tubulin-related proteins in (+)-PTC-treated PC-3 cells aligns with previous findings demonstrating the anti-mitotic effect of (+)-PTC in prostate cancer cells. This effect is characterized by impaired bipolar spindle formation and occurs without affecting the number of centrosomes and so reinforcing the notion that (+)-PTC disrupts cytoskeletal proteins dynamics (Moraes de Farias et al., 2022). The proteomic data presented here provide further evidence supporting earlier results and so strengthening the mechanistic understanding of (+)-PTC’s impact on cytoskeletal integrity and mitotic progression. Moreover, several studies have demonstrated that damage in microtubule networks affects not only mitotic cells but also interphase cells by disrupting different signaling pathways such as kinase activation and gene transcription (Bates and Eastman, 2017).

The destabilization of proteins other than tubulin has an enormous impact on cytoskeleton organization and in this context it is notable that (+)-PTC was able to affect (relative to the negative control) a group of proteins related to the cytoskeleton including those arising from the KRT16, KRT40, MYH3, MYH14, ACTN4 and SPTAN1 genes. This cluster of proteins is important in cytoskeletal organization and related to the actin organization (Aseervatham, 2020). For example, the keratins (*ex*. the KRT16 and KRT40 encoding genes) are part of intermediate filaments that play critical roles in protecting cells from different stressors as well as in cell motility, cell morphology and membrane trafficking (Sarma, 2022; Takan et al., 2023).

Notably, transketolase (TKT) and ALAS1, both critical for cancer cell metabolism and heme synthesis respectively, were undetectable in (+)-PTC-treated cells (Table 1). TKT over-expression correlates with chemoresistance and metastasis in multiple cancers while ALAS1 is essential for mitochondrial function (Hao et al., 2022; Hooda et al., 2013; Li et al., 2022; Xu et al., 2016; Cardama et al., 2017). The absence of both proteins suggests (+)-PTC may disrupt metabolic processes essential for prostate cancer cell survival beyond just cytoskeletal effects.

Reactome pathway analysis of the upregulated proteins observed in (+)-PTC-treated PC-3 cells revealed that these are mainly associated with pathways related to Rho GTPase-mediated processes, such enzymes being a set of proteins that alternate between inactive and active states (viz. GDP- and GTP-bound states, respectively), promoting the activation of effector proteins that, in turn, regulate cell shape, attachments, cell cycle progression, cell migration, proliferation and cytoskeletal organization (Haga and Ridley, 2016; Liu et al., 1998). During intrinsic or extrinsic stresses, such as exposure to anti-tubulin compounds, Rho GTPases and their associated proteins are activated so as to restore cytoskeletal integrity (Liu et al., 2020; Ohashi et al., 2017; Thompson et al., 2021). Among the proteins upregulated through (+)-PTC treatment were those arising from the DOCK2, PIK3CA and CIT genes, these being enzymes associated with Rho GTPase functions. The upregulation of Rho GTPase-associated proteins may represent a compensatory cellular response to cytoskeletal disruption.

The findings detailed above are hypothesis-generating and suggest that (+)-PTC exerts its anti-mitotic and cytotoxic effects by destabilizing cytoskeleton-related proteins leading to loss of MMP and intrinsic apoptosis. While preliminary selectivity data against PBMCs suggest a potential therapeutic window (selectivity index >6-fold; Moraes de Farias et al., 2022), these represent just one model for assessing normal cell toxicity and may not predict adverse drug effects in other clinically relevant tissues such as neurons. Clearly, then, comprehensive toxicity profiling in such cell types and direct comparative studies with, for example, standard-of-care taxanes is necessary to fully evaluate the therapeutic potential of (+)-PTC. Further, and given that the deep proteomic characterization reported here was only performed in PC-3 cells, and thus representing just one subtype of mCRPC, further validation in different mCRPC cell lines is necessary to round-out the current findings. Clearly, additional and orthogonal validation studies are required to fully assess the underlying origins of the key differentially expressed proteins (TTLL3, PRKCB, TKT, ALAS1, tubulin isoforms) observed in the proteomic assay as well as the development of a biochemical assay to establish the effective target. More generally, and to better understand its therapeutic potential, future studies should focus on assessing (+)-PTC in more physiologically-relevant models.

## 5 Conclusion

This study details a global proteomic analysis of a prostate cancer cell line treated with (+)-PTC and so revealing the ability of this natural product to alter the abundance of cytoskeleton-associated proteins that lead to loss of mitochondrial membrane polarization and apoptosis. Notably, this proteomic profile shows a partial overlap with that arising from treating the same cell line with the proven microtubule disrupting agent nocodazole. These findings add to the growing body of evidence supporting the notion that (+)-PTC acts as an anti-mitotic agent. As such, this work provides insights for future studies aimed at validating the compounds molecular target(s) as well as for assessing the therapeutic potential of (+)-PTC in preclinical models.

## Data Availability

The datasets presented in this study can be found in online repositories. The names of the repository/repositories and accession number(s) can be found below: https://www.ebi.ac.uk/pride/archive/, PXD073655.
