# Supplementary material for: The pterocarpan (+)-PTC modulates cytoskeletal proteins and induces apoptosis in metastatic castration-resistant prostate cancer: a proteomic perspective
Source: Front Pharmacol. 2026 Jun 3;17:1770249. doi: 10.3389/fphar.2026.1770249 (PMC13272309; doi:10.3389/fphar.2026.1770249)
Supplement: Supplementary file 1 [file Supplementaryfile1.docx]

The pterocarpan (+)-PTC modulates cytoskeletal proteins and induces apoptosis in metastatic castration-resistant prostate cancer:

a proteomic perspective.

José de Brito Vieira Neto^2,#,*^, Kaio Moraes de Farias^1,2,#^, Sarah Leyenne Alves Sales^2^, Vitoria Braga Melo^3^, Stella Maria Nascimento Macêdo^2,^ Carlos Roberto Koscky Paier^2^, Maria Júlia Bezerra^2,3^, Arlindo A. Moura^1,2,4^, Hernandes F. Carvalho^5^, Felipe Domingos de Sousa^12^, Adriano Aquino^7^, Ana Cristina Moreira^11^, Daniel Martins-de-Souza^7,8,9,10^, Martin G. Banwell^6^ , Cristiana Libardi Miranda Furtado^13,#,*^ and Claudia Pessoa^1,2,#,^.

In support of this manuscript, this section contains supplementary figures and tables relevant for the understanding of the article.

**Supplementary Table 1**. All DEPs observed in (+)-PTC-treated PC-3 cells

(compared to the negative control).

| Gene | Description | Anova (p) | logFC | stauts |
| --- | --- | --- | --- | --- |
| KDM2B | Lysine-specific demethylase 2B | 0.022930191 | -9.069387934 | down |
| PTOV1 | Prostate tumor-overexpressed gene 1 protein | 0.006475824 | -8.861643593 | down |
| YWHAQ | Tyrosine 3-monooxygenase/tryptophan 5-monooxygenase activation protein theta (Fragment) | 0.00083249 | -7.798678692 | down |
| PSMB6 | Proteasome subunit beta | 0.033734669 | -7.534033521 | down |
| DPP6 | Dipeptidyl aminopeptidase-like protein 6 | 0.002955575 | -7.052088697 | down |
| ANAPC7 | Anaphase-promoting complex subunit 7 | 0.003566738 | 5.67245772 | up |
| TTLL3 | Tubulin tyrosine ligase like 3 | 0.016539201 | 5.538039821 | up |
| SCARB2 | Scavenger receptor class B member 2 | 0.004998242 | -6.799340684 | down |
| UBE2D3 | Ubiquitin-conjugating enzyme E2 D3 | 0.01761282 | -6.785682072 | down |
| AP3B1 | Adaptor-related protein complex 3 beta 1 subunit isoform 1 (Fragment) | 0.00219816 | -6.681279843 | down |
| ARHGAP28 | Rho GTPase activating protein 28 | 0.000253022 | -6.663060556 | down |
| DDX11L8 | Putative ATP-dependent DNA helicase DDX11-like protein 8 | 0.000205105 | -6.64744264 | down |
| PRKCB | Protein kinase C beta (Fragment) | 0.001142472 | -6.28544065 | down |
| EPB41L1 | Erythrocyte membrane protein band 4.1 like 1 | 0.027665546 | -5.821332451 | down |
| PPFIA4 | Liprin-alpha-4 | 0.042091297 | -5.736140095 | down |
| CTU2 | Cytoplasmic tRNA 2-thiolation protein 2 | 0.000783129 | -5.427182399 | down |
| PIK3CA | phosphatidylinositol-4_5-bisphosphate 3-kinase | 0.036882103 | 4.013366075 | up |
| FSHD | Facioscapulohumeral muscular dystrophy | 0.000056 | -5.34690162 | down |
| SRP72 | SRP72 protein (Fragment) | 0.000930978 | -5.193310554 | down |
| TUBA3C | Tubulin alpha-3C chain | 0.002114856 | -5.157363128 | down |
| ARMC9 | LisH domain-containing protein ARMC9 | 0.009034678 | -5.098930426 | down |
| DSG2 | Desmoglein-2 | 0.005296667 | -5.086609016 | down |
| TAB3 | TGF-beta-activated kinase 1 and MAP3K7-binding protein 3 | 0.003077034 | -5.028000027 | down |
| EML5 | Echinoderm microtubule-associated protein-like 5 | 0.0000995 | -5.006406227 | down |
| SFN | 14-3-3 protein sigma | 0.002792903 | -4.986797639 | down |
| NPEPPS | Aminopeptidase | 0.020381843 | -4.870282825 | down |
| MYH14 | Myosin-14 | 0.004678989 | -4.764344018 | down |
| COL16A1 | Collagen alpha-1(XVI) chain | 0.002645833 | 3.343536839 | up |
| ARID4B | AT-rich interactive domain-containing protein 4B | 0.000553309 | 3.299765048 | up |
| ARHGAP21 | Rho GTPase activating protein 21 | 0.000646939 | -4.651014796 | down |
| ARHGEF2 | Rho guanine nucleotide exchange factor 2 | 0.013203532 | -4.569541459 | down |
| YBX1 | YBX1 protein (Fragment) | 0.003414008 | -4.562664838 | down |
| CLSPN | Claspin | 0.048186004 | 3.09186894 | up |
| ANGPTL4 | Angiopoietin like 4 (Fragment) | 0.021617638 | -4.548126518 | down |
| FAN1 | Fanconi-associated nuclease | 0.000547665 | -4.411569708 | down |
| MYLK | Myosin light chain kinase | 0.004038542 | 3.00072571 | up |
| CHCHD2 | Coiled-coil-helix-coiled-coil-helix domain-containing protein 2 | 0.001623714 | -4.366908954 | down |
| PAPLN | Papilin | 0.005468852 | 2.906920829 | up |
| PDE4A | 3'_5'-cyclic-AMP phosphodiesterase 4A | 0.007699696 | -4.298251384 | down |
| HNRNPDL | Heterogeneous nuclear ribonucleoprotein D like | 0.000535177 | -4.302995778 | down |
| UBR3 | E3 ubiquitin-protein ligase UBR3 | 0.006936542 | 2.919870861 | up |
| KIF5A | Kinesin family member 5A | 0.017176443 | -4.231251037 | down |
| FAM21A | FAM21A protein | 0.014987217 | 2.80575458 | up |
| RAD23A | UV excision repair protein RAD23 homolog A | 0.001156839 | -4.150296127 | down |
| ABCB4 | Phosphatidylcholine translocator ABCB4 | 0.00867303 | -4.139601998 | down |
| ARID1A | AT-rich interactive domain-containing protein 1A | 0.001592638 | 2.696512973 | up |
| ANK3 | Ankyrin 3 (Fragment) | 0.001280788 | -4.059302952 | down |
| HNRPA3 | Epididymis secretory sperm binding protein | 0.007778685 | 2.537104058 | up |
| DMPK | non-specific serine/threonine protein kinase | 0.02167626 | -3.805805207 | down |
| CSF1R | Platelet-derived growth factor receptor-like protein (Fragment) | 0.00000675 | -3.801365695 | down |
| CLEC7A | C-type lectin domain family 7 member A isoform 1 (Fragment) | 0.000609758 | 2.375726739 | up |
| NME1 | Nucleoside diphosphate kinase A | 0.000451617 | -3.754215984 | down |
| PABPC4 | Polyadenylate-binding protein | 0.024143181 | -3.749739224 | down |
| PSME2 | Proteasome activator complex subunit 2 | 0.003220078 | -3.666178014 | down |
| ANKRD36C | Ankyrin repeat domain 36C | 0.02952442 | -3.555476878 | down |
| RBM20 | RNA-binding protein 20 | 0.007309103 | 2.206418545 | up |
| KCNA10 | Potassium voltage-gated channel subfamily A member 10 | 0.004155831 | 2.153975468 | up |
| TENC1 | Tensin like C1 domain containing phosphatase (Tensin 2) | 0.02568547 | -3.535440325 | down |
| MMS19 | MMS19 nucleotide excision repair protein homolog | 0.001968511 | 2.1090133 | up |
| GPR85 | G protein-coupled receptor 85 | 0.006943082 | 2.122453697 | up |
| RAVER1 | Ribonucleoprotein PTB-binding 1 | 0.001946706 | -3.461933467 | down |
| U2AF2 | Splicing factor U2AF subunit | 0.014004084 | -3.46233383 | down |
| CFAP44 | Cilia- and flagella-associated protein 44 | 0.002236399 | 2.028809884 | up |
| PPA1 | Inorganic pyrophosphatase | 0.003556881 | -3.417123829 | down |
| AP2A1 | AP-2 complex subunit alpha-1 | 0.000122427 | -3.401883062 | down |
| FSTL5 | Follistatin-related protein 5 | 0.005981852 | -3.38899082 | down |
| GUK1 | guanylate kinase | 0.020777233 | -3.381584428 | down |
| CBL | E3 ubiquitin-protein ligase CBL | 0.001154237 | -3.317029265 | down |
| APC2 | Adenomatous polyposis coli protein 2 | 0.006850556 | -3.269363776 | down |
| FUT6 | 4-galactosyl-N-acetylglucosaminide 3-alpha-L-fucosyltransferase FUT6 | 0.011304037 | -3.221125732 | down |
| GON4L | GON4L protein (Fragment) | 0.003501594 | 1.826325498 | up |
| RAB3GAP2 | RAB3 GTPase activating non-catalytic protein subunit 2 | 0.01497852 | -3.20096964 | down |
| POTEF | POTE ankyrin domain family member F | 0.009004663 | -3.180729762 | down |
| PUF60 | Poly(U)-binding-splicing factor PUF60 | 0.007707506 | -3.212472482 | down |
| ANKMY1 | Ankyrin repeat and MYND domain containing 1 | 0.002218278 | -3.191068742 | down |
| OAS1 | 2'-5'-oligoadenylate synthetase 1 | 0.005215305 | -3.156402539 | down |
| CDHR1 | Cadherin-related family member 1 | 0.049114071 | -3.148141789 | down |
| DCDC1 | Doublecortin domain containing 1 | 0.02304881 | -3.09211686 | down |
| H2AX | Histone H2AX | 0.001313946 | -3.103315658 | down |
| MPRIP | Myosin phosphatase Rho interacting protein (Fragment) | 0.01265354 | 1.684967648 | up |
| CDK12 | Cyclin-dependent kinase 12 | 0.001045965 | -3.084067594 | down |
| PRX | Periaxin | 0.018485354 | 1.668490993 | up |
| CDK13 | Cyclin dependent kinase 13 (Fragment) | 0.003804024 | -3.062479842 | down |
| EIF2S3 | Eukaryotic translation initiation factor 2 subunit 3 | 0.004321867 | -3.04903478 | down |
| HEATR1 | HEAT repeat-containing protein 1 | 0.005936325 | -3.04074738 | down |
| AP2A2 | Adaptor related protein complex 2 subunit alpha 2 (Fragment) | 0.000432391 | -3.03690165 | down |
| SLC12A9 | Solute carrier family 12 member 9 (Fragment) | 0.003232538 | 1.628458881 | up |
| COL4A1 | Collagen alpha-1(IV) chain | 0.012023185 | -3.025209968 | down |
| NAV3 | Neuron navigator 3 | 0.032815027 | -3.020457262 | down |
| ERVK-21 | Endogenous retrovirus group K member 21 Env polyprotein | 0.014282932 | 1.598938827 | up |
| GREB1 | Protein GREB1 | 0.005413376 | 1.579806733 | up |
| LCORL | Ligand dependent nuclear receptor corepressor like | 0.036267197 | 1.536886446 | up |
| SNRNP200 | U5 small nuclear ribonucleoprotein 200 kDa helicase (Fragment) | 0.019581078 | -2.931669729 | down |
| POM121C | Nuclear envelope pore membrane protein POM 121C | 0.007973653 | 1.548863976 | up |
| MCM7 | DNA replication licensing factor MCM7 | 0.020686196 | -2.935743303 | down |
| NR3C1 | Glucocorticoid receptor | 0.000970388 | 1.502167908 | up |
| RPL12 | Large ribosomal subunit protein uL11 | 0.000977498 | -2.867855566 | down |
| EIF3S9 | Eukaryotic translation initiation factor 3 subunit B | 0.003254645 | 1.457473197 | up |
| TCF12 | Transcription factor 12 (Fragment) | 0.001866382 | 1.433147736 | up |
| RPL22 | Large ribosomal subunit protein eL22 | 0.0000532 | -2.821156235 | down |
| CIT | non-specific serine/threonine protein kinase (Fragment) | 0.00206488 | 1.406808992 | up |
| RAN | GTP-binding nuclear protein Ran | 0.035380348 | 1.371816004 | up |
| PHIP | Pleckstrin homology domain interacting protein | 0.001889174 | 1.392892229 | up |
| MPDZ | Multiple PDZ domain protein | 0.038524572 | -2.790925113 | down |
| SCN4A | Sodium channel protein type 4 subunit alpha | 0.00196654 | 1.374226241 | up |
| NPR2 | Guanylate cyclase | 0.014602057 | 1.318459871 | up |
| DOCK2 | DOCK2 (Fragment) | 0.008712563 | 1.307265279 | up |
| N4BP1 | NEDD4-binding protein 1 | 0.002969955 | 1.301210548 | up |
| PYCR2 | Pyrroline-5-carboxylate reductase 2 | 0.003892514 | -2.695346428 | down |
| TPP2 | Tripeptidyl-peptidase 2 (Fragment) | 0.010895852 | -2.582569838 | down |
| MYO5A | Myosin VA | 0.003408143 | 1.14585381 | up |
| COL6A6 | Collagen alpha-6(VI) chain | 0.002960869 | -2.547999987 | down |
| RTTN | Rotatin (Fragment) | 0.001437504 | 1.143971106 | up |
| HLA-A | MHC class I antigen | 0.000139707 | -2.521602429 | down |
| CACNB4 | Calcium voltage-gated channel auxiliary subunit beta 4 (Fragment) | 0.011623896 | 1.123919262 | up |
| EPHA6 | Ephrin type-A receptor 6 | 0.006327993 | 1.093520244 | up |
| FLT1 | Vascular endothelial growth factor receptor 1 | 0.002828837 | 1.097800436 | up |
| ZC3H13 | Zinc finger CCCH-type containing 13 | 0.002049669 | -2.45395575 | down |
| ZNF516 | Zinc finger protein 516 | 0.010604637 | -2.454563127 | down |
| MIF | Macrophage migration inhibitory factor (Fragment) | 0.011135618 | -2.435097807 | down |
| GARRE1 | Granule associated Rac and RHOG effector 1 (Fragment) | 0.01851565 | 0.985052683 | up |
| GRID2 | Glutamate ionotropic receptor delta type subunit 2 (Fragment) | 0.018306185 | -2.388865057 | down |
| KRT16 | Keratin_ type I cytoskeletal 16 | 0.017878224 | -2.384705489 | down |
| SIN3A | SIN3 transcription regulator family member A | 0.010353711 | -2.374059527 | down |
| SPTAN1 | Spectrin alpha_ non-erythrocytic 1 (Fragment) | 0.013692559 | 0.96721497 | up |
| KAT6A | Histone acetyltransferase (Fragment) | 0.046373697 | 0.898190345 | up |
| NME2 | Nucleoside diphosphate kinase B | 0.000473594 | -2.29344523 | down |
| PSMD2 | 26S proteasome non-ATPase regulatory subunit 2 | 0.004281034 | -2.285746373 | down |
| PRDX5 | Peroxiredoxin-5_ mitochondrial | 0.001907794 | -2.231444747 | down |
| FRMPD4 | FERM and PDZ domain containing 4 | 0.014269919 | 0.819760985 | up |
| POTEE | POTE ankyrin domain family member E | 0.006159249 | 0.806146952 | up |
| ZNF717 | Zinc finger protein 717 | 0.003799796 | 0.801699987 | up |
| CGN | Cingulin | 0.016377598 | 0.789411585 | up |
| NES | Nestin | 0.002941541 | 0.786524856 | up |
| SCRIB | Protein scribble homolog | 0.030382174 | 0.792881277 | up |
| VSX2 | Visual system homeobox 2 | 0.004356851 | 0.78811687 | up |
| PHB | Prohibitin | 0.040315552 | -2.178823756 | down |
| MEF2D | Myocyte-specific enhancer factor 2D | 0.027557341 | 0.765782643 | up |
| SNX29 | Sorting nexin 29 (Fragment) | 0.013411501 | 0.760445659 | up |
| DNMT1 | DNA (cytosine-5)-methyltransferase | 0.010297237 | 0.755171362 | up |
| N4BP2 | NEDD4-binding protein 2 | 0.00136229 | 0.737969534 | up |
| COX2 | Cytochrome c oxidase subunit 2 | 0.024532329 | 0.72594262 | up |
| TUBGCP5 | Gamma-tubulin complex component | 0.007520038 | 0.713357034 | up |
| PTPRS | Receptor-type tyrosine-protein phosphatase S | 0.006700506 | 0.690749502 | up |
| ZNF582 | Zinc finger protein 582 | 0.020737304 | -2.088118721 | down |
| FLJ00119 | Epididymis secretory sperm binding protein (Fragment) | 0.009963654 | -2.081870044 | down |
| EPB41L3 | Erythrocyte membrane protein band 4.1 like 3 | 0.019003893 | 0.661530522 | up |
| DNM1L | Dynamin-1-like protein | 0.01150735 | 0.652716683 | up |
| GCC2 | GRIP and coiled-coil domain containing 2 | 0.00539483 | -2.043693644 | down |
| NEBL | Nebulette variant 4 | 0.001374087 | 0.639102793 | up |
| SF3B1 | SF3B1 protein (Fragment) | 0.024864342 | 0.629731244 | up |
| BAZ2A | Bromodomain adjacent to zinc finger domain 2A | 0.01550101 | -2.011569234 | down |
| SPATS2L | SPATS2-like protein | 0.04151793 | 0.597627712 | up |
| CUX1 | Homeobox protein cut-like | 0.008299561 | 0.618614975 | up |
| LARP1 | La ribonucleoprotein 1_ translational regulator | 0.002639943 | 0.607507586 | up |
| RNF20 | E3 ubiquitin protein ligase (Fragment) | 0.018601214 | -1.978853355 | down |
| OR14J1 | Olfactory receptor | 0.009921688 | 0.600093945 | up |
| TNRC6A | Trinucleotide repeat containing adaptor 6A (Fragment) | 0.003027053 | -1.991331454 | down |
| ECT2 | Protein ECT2 | 0.032098745 | 0.594923603 | up |
| PTGES3 | Prostaglandin E synthase 3 | 0.009277911 | -1.982060709 | down |
| G3BP2 | Ras GTPase-activating protein-binding protein 2 | 0.001307843 | -1.975263823 | down |
| RBM3 | RNA-binding protein 3 | 0.000737536 | 0.58403726 | up |
| BAZ2B | Bromodomain adjacent to zinc finger domain protein 2B | 0.001784936 | -1.97636206 | down |
| RPS5 | Small ribosomal subunit protein uS7 (Fragment) | 0.00492749 | -1.957040797 | down |
| KRT40 | Keratin 40 | 0.047713764 | -1.948039236 | down |
| NONO | Non-POU domain containing octamer-binding isoform 1 (Fragment) | 0.038053315 | -1.907675346 | down |
| DDX46 | RNA helicase | 0.006331327 | -1.857792653 | down |
| MIA2 | MIA SH3 domain ER export factor 2 | 0.002591551 | -1.852853222 | down |
| ZNF431 | Zinc finger protein 431 (Fragment) | 0.033874525 | -1.849123418 | down |
| F5-20 | F5-20 (Fragment) | 0.000581706 | -1.836111937 | down |
| PDE8A | High affinity cAMP-specific and IBMX-insensitive 3'_5'-cyclic phosphodiesterase 8A | 0.010643051 | -1.813046437 | down |
| GAPVD1 | GTPase activating protein and VPS9 domains 1 | 0.004472951 | -1.805024333 | down |
| EIF4A2 | Eukaryotic initiation factor 4A-II | 0.044345868 | -1.808615072 | down |
| ABL1 | Tyrosine-protein kinase ABL1 | 0.002261918 | -1.7774806 | down |
| MAP1B | MAP1B protein (Fragment) | 0.015522007 | -1.744978135 | down |
| CCDC30 | Coiled-coil domain containing 30 | 0.010741064 | -1.726184548 | down |
| RPLP2 | Large ribosomal subunit protein P2 | 0.010874151 | -1.732267154 | down |
| RPLP1 | Large ribosomal subunit protein P1 | 0.00472042 | -1.682915412 | down |
| TUBA1B | Tubulin alpha-1B chain | 0.021169258 | -1.681138214 | down |
| CFL1 | Cofilin-1 | 0.007996817 | -1.676912933 | down |
| NRXN3 | Neurexin 3 | 0.024341364 | -1.677945075 | down |
| TOGARAM1 | KIAA0423_ isoform CRA_a | 0.020998925 | -1.667788212 | down |
| ACTN4 | Alpha-actinin-4 (Fragment) | 0.01219901 | -1.666031858 | down |
| YWHAE/FAM22A fusion | 14-3-3 protein epsilon (Fragment) | 0.022060407 | -1.6648752 | down |
| HLA-DQB1 | MHC class II antigen (Fragment) | 0.019694046 | -1.657598031 | down |
| GART | Trifunctional purine biosynthetic protein adenosine-3 | 0.002575672 | -1.646639445 | down |
| DOCK7 | Dedicator of cytokinesis protein 7 | 0.015220045 | -1.63548715 | down |
| ZSCAN26 | Zinc finger and SCAN domain containing 26 | 0.018309781 | -1.580616657 | down |
| TUBA4A | Tubulin alpha-4A chain | 0.009748902 | -1.587634577 | down |
| NCOA2 | Nuclear receptor coactivator 2 (Fragment) | 0.025062511 | -1.578247261 | down |
| LDHA | L-lactate dehydrogenase A chain | 0.002375742 | -1.575047843 | down |
| IFT172 | Intraflagellar transport 172 | 0.004383754 | -1.54233516 | down |
| AOC3 | Amine oxidase [copper-containing] 3 | 0.008012 | -1.518836129 | down |
| ENO1 | Alpha-enolase | 0.013593186 | -1.479286572 | down |
| RPS7 | 40S ribosomal protein S7 | 0.044146593 | -1.466664616 | down |
| CFL2 | Cofilin-2 | 0.011327161 | -1.420296726 | down |
| MYH3 | Myosin-3 | 0.015673901 | -1.418676155 | down |
| MEI1 | Meiosis inhibitor protein 1 | 0.039183627 | -1.406718823 | down |
| KAT8 | Histone acetyltransferase KAT8 | 0.008050151 | -1.394841361 | down |
| MAPK4 | mitogen-activated protein kinase | 0.015798452 | -1.385082235 | down |
| PFN1 | Profilin-1 | 0.003340978 | -1.371722972 | down |
| ZNF233 | Zinc finger protein 233 | 0.003977176 | -1.354129124 | down |
| CTNND2 | Catenin delta 2 | 0.018294298 | -1.352839 | down |
| TUBB8B | Tubulin beta 8B | 0.042913172 | -1.342162534 | down |
| NINL | Ninein-like protein | 0.035486229 | -1.340421318 | down |
| EEF1A2 | Elongation factor 1-alpha | 0.02781941 | -1.342917806 | down |
| CCDC88C | Protein Daple | 0.026604856 | -1.327486539 | down |
| PGK1 | Phosphoglycerate kinase 1 | 0.006717774 | -1.314690544 | down |
| HNRNPU | Heterogeneous nuclear ribonucleoprotein U | 0.039860655 | -1.307745228 | down |
| RACK1 | Small ribosomal subunit protein RACK1 | 0.020101282 | -1.301824681 | down |
| PREX2 | PREX2 (Fragment) | 0.000285759 | -1.292802214 | down |
| MATR3 | Matrin-3 | 0.028812221 | -1.28770654 | down |

**Supplementary Table 2.** All DEPs observed in Nocodazol-treated PC-3 cells

(compared to the negative control).

| Gene Name | Description | pValue | logFC | status |
| --- | --- | --- | --- | --- |
| CHCHD2 | Coiled-coil-helix-coiled-coil-helix domain-containing protein 2 | 0.0000963 | -4.593661719 | down |
| CDK12 | Cyclin-dependent kinase 12 | 0.0000995 | -3.869952664 | down |
| TNRC6A | Trinucleotide repeat containing adaptor 6A (Fragment) | 0.000114816 | -2.412822709 | down |
| KDM2B | Lysine-specific demethylase 2B | 0.000145761 | -10.48859341 | down |
| CTNNA1 | Catenin alpha-1 | 0.000183881 | 3.284492105 | up |
| FAN1 | Fanconi-associated nuclease | 0.000213901 | -4.226101141 | down |
| PUF60 | Poly(U)-binding-splicing factor PUF60 | 0.000274243 | -4.301982009 | down |
| TUBA3C | Tubulin alpha-3C chain | 0.000312932 | -5.779010671 | down |
| CLEC7A | C-type lectin domain family 7 member A isoform 1 (Fragment) | 0.000338165 | 2.936787355 | up |
| HLA-A | MHC class I antigen | 0.000376301 | -3.799106589 | down |
| NPEPPS | Aminopeptidase | 0.000376377 | -4.730181511 | down |
| YWHAQ | Tyrosine 3-monooxygenase/tryptophan 5-monooxygenase activation protein theta (Fragment) | 0.000389078 | -8.040551814 | down |
| ARID4B | AT-rich interactive domain-containing protein 4B | 0.000396545 | 3.730328908 | up |
| AP2A1 | AP-2 complex subunit alpha-1 | 0.000495566 | -3.371039728 | down |
| MEI1 | Meiosis inhibitor protein 1 | 0.000535985 | -1.814860502 | down |
| SRP72 | SRP72 protein (Fragment) | 0.000550959 | -6.745367265 | down |
| MMS19 | MMS19 nucleotide excision repair protein homolog | 0.00055795 | 2.345215463 | up |
| ZNF582 | Zinc finger protein 582 | 0.000584989 | -2.3604081 | down |
| MYO5A | Myosin VA | 0.000614223 | 1.07170899 | up |
| YBX1 | YBX1 protein (Fragment) | 0.000632336 | -4.539102078 | down |
| N4BP1 | NEDD4-binding protein 1 | 0.000676751 | 1.213129897 | up |
| ABCB4 | Phosphatidylcholine translocator ABCB4 | 0.000679395 | -4.513870787 | down |
| COL6A6 | Collagen alpha-6(VI) chain | 0.000715791 | -3.000550049 | down |
| GREB1 | Protein GREB1 | 0.00072745 | 3.19140897 | up |
| BAZ2B | Bromodomain adjacent to zinc finger domain protein 2B | 0.000759975 | -1.784315238 | down |
| MYH14 | Myosin-14 | 0.000840501 | -5.508872614 | down |
| RAVER1 | Ribonucleoprotein PTB-binding 1 | 0.000841645 | -3.449002529 | down |
| ARHGAP21 | Rho GTPase activating protein 21 | 0.000875246 | -7.097374511 | down |
| GAN | Epididymis secretory sperm binding protein (Fragment) | 0.000892896 | 0.713025099 | up |
| H2AX | Histone H2AX | 0.000986091 | -3.90962729 | down |
| FLT1 | Vascular endothelial growth factor receptor 1 | 0.001030441 | 1.146884861 | up |
| SHANK3 | SH3 and multiple ankyrin repeat domains 3 | 0.001052239 | 1.209409866 | up |
| DSG2 | Desmoglein-2 | 0.001101012 | -4.874372389 | down |
| NAV3 | Neuron navigator 3 | 0.001225634 | -3.229005662 | down |
| CFL2 | Cofilin-2 | 0.001230163 | -1.722421901 | down |
| CALR | Calreticulin | 0.001300215 | -1.95785856 | down |
| CTNND2 | Catenin delta 2 | 0.001310079 | -1.342258874 | down |
| MIF | Macrophage migration inhibitory factor (Fragment) | 0.001328428 | -3.167049873 | down |
| PABPC4 | Polyadenylate-binding protein | 0.001386545 | -4.839498145 | down |
| AOC3 | Amine oxidase [copper-containing] 3 | 0.001419173 | -1.80616764 | down |
| RPL22 | Large ribosomal subunit protein eL22 | 0.001422221 | -3.279533427 | down |
| GART | Trifunctional purine biosynthetic protein adenosine-3 | 0.001426175 | -2.027744803 | down |
| CDHR1 | Cadherin-related family member 1 | 0.00144004 | -6.547756676 | down |
| PGK1 | Phosphoglycerate kinase 1 | 0.001621271 | -1.32692256 | down |
| PLXND1 | Plexin-D1 | 0.001662623 | -2.043492434 | down |
| RPL12 | Large ribosomal subunit protein uL11 | 0.001711772 | -2.915363817 | down |
| PSME2 | Proteasome activator complex subunit 2 | 0.001722775 | -6.120750101 | down |
| ZNF233 | Zinc finger protein 233 | 0.001727891 | -1.461452404 | down |
| COL4A1 | Collagen alpha-1(IV) chain | 0.001739454 | -3.361250142 | down |
| RNPC3 | RNA-binding region-containing protein 3 | 0.00180949 | -1.917976123 | down |
| EIF3S9 | Eukaryotic translation initiation factor 3 subunit B | 0.001817321 | 2.464459396 | up |
| NME1 | Nucleoside diphosphate kinase A | 0.001909211 | -3.800790883 | down |
| LYST | Lysosomal trafficking regulator (Fragment) | 0.001967521 | -2.178333865 | down |
| CFL1 | Cofilin-1 | 0.001984473 | -1.820838316 | down |
| PLXNB2 | Plexin-B2 | 0.002035695 | -2.490883951 | down |
| ZNF717 | Zinc finger protein 717 | 0.002118366 | 0.986511853 | up |
| TCF12 | Transcription factor 12 (Fragment) | 0.002181393 | 1.88978162 | up |
| DDX46 | RNA helicase | 0.002212798 | -2.17090118 | down |
| BRD4 | Bromodomain containing 4 | 0.002289752 | -1.393985941 | down |
| ARID1A | AT-rich interactive domain-containing protein 1A | 0.002307617 | 1.639049606 | up |
| HEL-S-284 | Aconitate hydratase_ mitochondrial | 0.002378962 | -4.610211258 | down |
| FSTL5 | Follistatin-related protein 5 | 0.002397851 | -4.018402008 | down |
| RTTN | Rotatin (Fragment) | 0.002504443 | 1.41862779 | up |
| AP2A2 | Adaptor related protein complex 2 subunit alpha 2 (Fragment) | 0.002613487 | -3.072518371 | down |
| PFN1 | Profilin-1 | 0.002799204 | -1.343935032 | down |
| POTEE | POTE ankyrin domain family member E | 0.003038191 | 0.911451545 | up |
| MAP4 | Microtubule-associated protein | 0.003303617 | -2.23024968 | down |
| PAK1 | Serine/threonine-protein kinase PAK 1 | 0.003503168 | 0.955816334 | up |
| GRID2 | Glutamate ionotropic receptor delta type subunit 2 (Fragment) | 0.003596187 | -3.664672913 | down |
| NME2 | Nucleoside diphosphate kinase B | 0.003607936 | -2.541330462 | down |
| CACNB4 | Calcium voltage-gated channel auxiliary subunit beta 4 (Fragment) | 0.003646116 | 1.458342852 | up |
| FGFR3 | Fibroblast growth factor receptor | 0.003842129 | -1.621574689 | down |
| SATB2 | SATB homeobox 2 (Fragment) | 0.003958326 | -3.210358048 | down |
| RPS5 | Small ribosomal subunit protein uS7 (Fragment) | 0.004049868 | -2.131955309 | down |
| MIA2 | MIA SH3 domain ER export factor 2 | 0.004176188 | -2.255197649 | down |
| RAD23A | UV excision repair protein RAD23 homolog A | 0.004281041 | -3.510897623 | down |
| ABL1 | Tyrosine-protein kinase ABL1 | 0.004437263 | -1.857030045 | down |
| FLJ00119 | Epididymis secretory sperm binding protein (Fragment) | 0.004505431 | -2.214378407 | down |
| GPR85 | G protein-coupled receptor 85 | 0.004582168 | 2.421774623 | up |
| PRKCB | Protein kinase C beta (Fragment) | 0.00474366 | -8.778161656 | down |
| EEF1A1P5 | Putative elongation factor 1-alpha-like 3 | 0.004753418 | -1.582291176 | down |
| SLC12A9 | Solute carrier family 12 member 9 (Fragment) | 0.004787844 | 1.488571104 | up |
| GON4L | GON4L protein (Fragment) | 0.004822189 | 1.180470398 | up |
| SFN | 14-3-3 protein sigma | 0.004850294 | -6.68670609 | down |
| ZNF516 | Zinc finger protein 516 | 0.004990442 | -2.533828598 | down |
| GUK1 | guanylate kinase | 0.005025594 | -3.598364034 | down |
| PTOV1 | Prostate tumor-overexpressed gene 1 protein | 0.005045589 | -7.023713687 | down |
| IFT172 | Intraflagellar transport 172 | 0.005186156 | -1.982298149 | down |
| RPLP2 | Large ribosomal subunit protein P2 | 0.005278267 | -1.886647476 | down |
| DCDC1 | Doublecortin domain containing 1 | 0.005445086 | -3.647936919 | down |
| PRDX5 | Peroxiredoxin-5_ mitochondrial | 0.005469789 | -2.601323 | down |
| SIN3A | SIN3 transcription regulator family member A | 0.005494165 | -4.479093808 | down |
| RBM20 | RNA-binding protein 20 | 0.005500253 | 2.577186214 | up |
| RPS7 | 40S ribosomal protein S7 | 0.005728248 | -1.445223511 | down |
| NR3C1 | Glucocorticoid receptor | 0.005771456 | 1.631567693 | up |
| NCOA2 | Nuclear receptor coactivator 2 (Fragment) | 0.005813658 | -2.967191878 | down |
| CFAP44 | Cilia- and flagella-associated protein 44 | 0.005831933 | 1.566500149 | up |
| TUBA4A | Tubulin alpha-4A chain | 0.005874313 | -1.729536649 | down |
| TUBA1B | Tubulin alpha-1B chain | 0.00597548 | -1.964857645 | down |
| MYLK | Myosin light chain kinase | 0.006166362 | 2.149872869 | up |
| LDHA | L-lactate dehydrogenase A chain | 0.006190101 | -1.796414823 | down |
| ACTN4 | Alpha-actinin-4 (Fragment) | 0.00630321 | -1.723981831 | down |
| PDE8A | High affinity cAMP-specific and IBMX-insensitive 3'_5'-cyclic phosphodiesterase 8A | 0.006318631 | -2.009831585 | down |
| BAZ2A | Bromodomain adjacent to zinc finger domain 2A | 0.006378241 | -1.874491694 | down |
| CIT | non-specific serine/threonine protein kinase (Fragment) | 0.006540815 | 1.779876732 | up |
| HEL-S-297 | SH3 domain-binding glutamic acid-rich-like protein 3 | 0.006544694 | -5.157702887 | down |
| PTPRS | Receptor-type tyrosine-protein phosphatase S | 0.006626334 | 1.526182579 | up |
| DDX11L8 | Putative ATP-dependent DNA helicase DDX11-like protein 8 | 0.006814442 | -6.424670284 | down |
| COX2 | Cytochrome c oxidase subunit 2 | 0.007049114 | 0.678042724 | up |
| COL16A1 | Collagen alpha-1(XVI) chain | 0.007261864 | 3.602934184 | up |
| OAS1 | 2'-5'-oligoadenylate synthetase 1 | 0.007639158 | -3.974253817 | down |
| OR14J1 | Olfactory receptor | 0.007706106 | 0.986903313 | up |
| ADAM28 | ADAM metallopeptidase domain 28 | 0.007939114 | -1.723797957 | down |
| DOCK2 | DOCK2 (Fragment) | 0.007944625 | 1.504020181 | up |
| RPLP1 | Large ribosomal subunit protein P1 | 0.008008907 | -1.798316463 | down |
| HNRPA3 | Epididymis secretory sperm binding protein | 0.008041398 | 2.875472814 | up |
| ALDOA | Fructose-bisphosphate aldolase A | 0.008096799 | -1.287878443 | down |
| CCDC88A | Coiled-coil domain containing 88A | 0.008204305 | -1.384134171 | down |
| PLS3 | Plastin-3 | 0.008222055 | -1.619176281 | down |
| GRIPAP1 | GRIP1 associated protein 1 | 0.008366046 | 0.887843485 | up |
| GAPVD1 | GTPase activating protein and VPS9 domains 1 | 0.008395816 | -2.592866204 | down |
| ENO1 | Alpha-enolase | 0.008529153 | -1.510734832 | down |
| MATR3 | Matrin-3 | 0.00854201 | -1.464885496 | down |
| EPHA6 | Ephrin type-A receptor 6 | 0.008564538 | 0.981570783 | up |
| NFIL3 | Nuclear factor interleukin-3-regulated protein | 0.008626006 | -1.914304599 | down |
| KCNA10 | Potassium voltage-gated channel subfamily A member 10 | 0.008710447 | 2.467629115 | up |
| NES | Nestin | 0.008908584 | 0.763052039 | up |
| CLTCL1 | Clathrin heavy chain 2 | 0.008919168 | -1.76935173 | down |
| MYH3 | Myosin-3 | 0.009118948 | -1.82508675 | down |
| SCN4A | Sodium channel protein type 4 subunit alpha | 0.009161312 | 1.079175257 | up |
| TENC1 | Tensin like C1 domain containing phosphatase (Tensin 2) | 0.009317613 | -6.644211454 | down |
| MAP1B | MAP1B protein (Fragment) | 0.009669532 | -2.263101485 | down |
| TAB3 | TGF-beta-activated kinase 1 and MAP3K7-binding protein 3 | 0.009738698 | -7.562871364 | down |
| POTEF | POTE ankyrin domain family member F | 0.010011291 | -2.752802968 | down |
| KAT8 | Histone acetyltransferase KAT8 | 0.010353085 | -1.510564531 | down |
| ABCB1 | ATP-binding cassette_ sub-family B (MDR/TAP)_ member 1 | 0.010356982 | -1.363588049 | down |
| PDE4A | 3'_5'-cyclic-AMP phosphodiesterase 4A | 0.010387959 | -4.103862564 | down |
| EIF2S3 | Eukaryotic translation initiation factor 2 subunit 3 | 0.010411092 | -2.904503787 | down |
| RAB3GAP2 | RAB3 GTPase activating non-catalytic protein subunit 2 | 0.010627069 | -4.077543555 | down |
| RASGRP2 | RAS guanyl releasing protein 2 (Fragment) | 0.010824575 | -1.819562494 | down |
| PHB | Prohibitin | 0.010844264 | -3.237390722 | down |
| H2AC4 | Histone H2A type 1-B/E | 0.010978039 | -1.33902336 | down |
| KALRN | Kalirin RhoGEF kinase | 0.011039017 | -1.513495336 | down |
| MPDZ | Multiple PDZ domain protein | 0.011132632 | -3.39612566 | down |
| EEF2 | Elongation factor 2 | 0.011241092 | -1.779102028 | down |
| CDH7 | Cadherin-7 | 0.011371329 | 0.795196707 | up |
| MGRN1 | E3 ubiquitin-protein ligase (Fragment) | 0.012356084 | -1.302215052 | down |
| MTUS2 | Microtubule-associated tumor suppressor candidate 2 | 0.012784289 | -1.297945953 | down |
| NONO | Non-POU domain containing octamer-binding isoform 1 (Fragment) | 0.012792354 | -2.072786923 | down |
| PTGES3 | Prostaglandin E synthase 3 | 0.012897883 | -1.840837033 | down |
| MAPK4 | mitogen-activated protein kinase | 0.012982445 | -1.904390729 | down |
| TPP2 | Tripeptidyl-peptidase 2 (Fragment) | 0.013045454 | -2.270908295 | down |
| KIF5A | Kinesin family member 5A | 0.013579048 | -4.300807744 | down |
| ANKMY1 | Ankyrin repeat and MYND domain containing 1 | 0.013646156 | -3.886681763 | down |
| HNRNPU | Heterogeneous nuclear ribonucleoprotein U | 0.013649385 | -1.638297973 | down |
| PAPLN | Papilin | 0.013721788 | 1.619871895 | up |
| KRT16 | Keratin_ type I cytoskeletal 16 | 0.013792614 | -2.936575606 | down |
| CDC42BPB | non-specific serine/threonine protein kinase | 0.01388428 | -1.377971799 | down |
| ANAPC7 | Anaphase-promoting complex subunit 7 | 0.014222222 | 5.370291315 | up |
| ADCY2 | Adenylate cyclase type 2 | 0.014275119 | -1.706099056 | down |
| H3C15 | Histone H3.2 | 0.014533792 | -1.913740433 | down |
| LCORL | Ligand dependent nuclear receptor corepressor like | 0.014547101 | 1.366842565 | up |
| SCRIB | Protein scribble homolog | 0.014723151 | 1.078576004 | up |
| CSF1R | Platelet-derived growth factor receptor-like protein (Fragment) | 0.014743809 | -4.085447509 | down |
| TRIM28 | Transcription intermediary factor 1-beta | 0.015210552 | -1.730765145 | down |
| TET2 | Methylcytosine dioxygenase TET | 0.015314414 | 0.773136939 | up |
| FSHD | Facioscapulohumeral muscular dystrophy | 0.015717348 | -5.120624589 | down |
| PLEKHH3 | Pleckstrin homology domain-containing family H member 3 | 0.015802743 | 4.448864258 | up |
| CDK13 | Cyclin dependent kinase 13 (Fragment) | 0.01583194 | -3.115856893 | down |
| NRXN3 | Neurexin 3 | 0.015833215 | -1.744454087 | down |
| LOC124905564 | Uncharacterized protein | 0.015859656 | -1.414384205 | down |
| POLRMT | DNA-directed RNA polymerase_ mitochondrial | 0.016437423 | -2.07301214 | down |
| ERVK-21 | Endogenous retrovirus group K member 21 Env polyprotein | 0.016474637 | 1.372611974 | up |
| TTLL3 | Tubulin tyrosine ligase like 3 | 0.016619995 | 5.493289333 | up |
| KRT40 | Keratin 40 | 0.016644483 | -2.213542119 | down |
| ANGPTL4 | Angiopoietin like 4 (Fragment) | 0.017142005 | -6.15111235 | down |
| LMO7 | LIM domain 7 (Fragment) | 0.017346261 | -1.384079476 | down |
| POM121C | Nuclear envelope pore membrane protein POM 121C | 0.017393696 | 0.87333994 | up |
| FAM21A | FAM21A protein | 0.017756773 | 1.809899602 | up |
| TOGARAM1 | KIAA0423_ isoform CRA_a | 0.018001155 | -2.205159435 | down |
| LAMA4 | Laminin subunit alpha 4 | 0.018070732 | -1.603361208 | down |
| HNRNPDL | Heterogeneous nuclear ribonucleoprotein D like | 0.018353287 | -3.662644426 | down |
| BRCA1 | Breast cancer type 1 susceptibility protein | 0.018454604 | 0.64388606 | up |
| PHIP | Pleckstrin homology domain interacting protein | 0.018578461 | 0.598966392 | up |
| G3BP2 | Ras GTPase-activating protein-binding protein 2 | 0.019324935 | -2.22141551 | down |
| ANK3 | Ankyrin 3 (Fragment) | 0.019434554 | -4.221193961 | down |
| TUBB6 | Tubulin beta chain | 0.019477374 | -1.394058768 | down |
| MPRIP | Myosin phosphatase Rho interacting protein (Fragment) | 0.019637205 | 1.107858957 | up |
| HLA-DQB1 | MHC class II antigen (Fragment) | 0.019984208 | -1.540081393 | down |
| H3F3B | Histone H3 | 0.020301871 | -1.723112931 | down |
| H3C1 | Histone H3.1 | 0.020341975 | -2.225145323 | down |
| CADPS | Calcium dependent secretion activator | 0.020848951 | 1.093245044 | up |
| PSMD2 | 26S proteasome non-ATPase regulatory subunit 2 | 0.020860034 | -1.929277834 | down |
| UBE2O | (E3-independent) E2 ubiquitin-conjugating enzyme | 0.021275052 | -2.16182658 | down |
| SPIN2A | Spindlin family member 2A | 0.021753843 | -5.843600476 | down |
| YWHAE/FAM22A fusion | 14-3-3 protein epsilon (Fragment) | 0.021948876 | -1.727422445 | down |
| DHX9 | ATP-dependent RNA helicase A | 0.02214568 | -1.677545724 | down |
| MCM7 | DNA replication licensing factor MCM7 | 0.022568042 | -2.557168283 | down |
| PRX | Periaxin | 0.022598645 | 1.386619167 | up |
| FUT6 | 4-galactosyl-N-acetylglucosaminide 3-alpha-L-fucosyltransferase FUT6 | 0.024245432 | -5.188661708 | down |
| EIF4G1 | Eukaryotic translation initiation factor 4 gamma 1 | 0.024584205 | -8.54663 | down |
| DOCK7 | Dedicator of cytokinesis protein 7 | 0.024665628 | -1.949460325 | down |
| GPI | Glucose-6-phosphate isomerase (Fragment) | 0.02501455 | -1.511103503 | down |
| MYO9A | Myosin IXA (Fragment) | 0.025114697 | -5.88130808 | down |
| APC2 | Adenomatous polyposis coli protein 2 | 0.025369356 | -2.908448894 | down |
| SBF2 | SET binding factor 2 | 0.02570035 | -1.8299564 | down |
| ZNF667 | Zinc finger protein 667 | 0.025773071 | 3.41445337 | up |
| CTU2 | Cytoplasmic tRNA 2-thiolation protein 2 | 0.025903222 | -6.410810675 | down |
| CBL | E3 ubiquitin-protein ligase CBL | 0.026129693 | -3.33005737 | down |
| HLA-DPB1 | MHC class II antigen (Fragment) | 0.026389071 | -1.289834547 | down |
| MKX | Homeobox protein Mohawk | 0.026717056 | -4.0336637 | down |
| DMPK | non-specific serine/threonine protein kinase | 0.026727489 | -3.823099074 | down |
| DNMT1 | DNA (cytosine-5)-methyltransferase | 0.027997793 | 0.744638902 | up |
| UBA6 | Ubiquitin-like modifier-activating enzyme 6 | 0.028645327 | 0.907546018 | up |
| PDLIM1 | PDZ and LIM domain protein 1 | 0.02872237 | -4.610214682 | down |
| EIF4A2 | Eukaryotic initiation factor 4A-II | 0.028909374 | -1.506241473 | down |
| RAN | GTP-binding nuclear protein Ran | 0.029046467 | 1.476560717 | up |
| ZC3H13 | Zinc finger CCCH-type containing 13 | 0.030037347 | -2.384691437 | down |
| TUT4 | RNA uridylyltransferase | 0.030782506 | -3.210894217 | down |
| TUBB8B | Tubulin beta 8B | 0.032125824 | -1.432376328 | down |
| H1-4 | Histone H1.4 | 0.032247813 | -1.329320305 | down |
| EEF1A2 | Elongation factor 1-alpha | 0.033680883 | -1.638953374 | down |
| SPTAN1 | Spectrin alpha_ non-erythrocytic 1 (Fragment) | 0.033762149 | 0.635977519 | up |
| PIK3R1 | Phosphatidylinositol 3-kinase regulatory subunit alpha (Fragment) | 0.033793175 | 11.35607056 | up |
| NT5DC3 | 5'-nucleotidase domain-containing protein 3 | 0.03409387 | -4.249197434 | down |
| HEATR1 | HEAT repeat-containing protein 1 | 0.034211309 | -3.02865452 | down |
| PIK3CA | phosphatidylinositol-4_5-bisphosphate 3-kinase | 0.034419237 | 4.197691905 | up |
| PLEKHG6 | PLEKHG6 (Fragment) | 0.035490414 | -3.059176614 | down |
| UBR3 | E3 ubiquitin-protein ligase UBR3 | 0.037317504 | 2.467596829 | up |
| GARRE1 | Granule associated Rac and RHOG effector 1 (Fragment) | 0.038873137 | 0.712457145 | up |
| TOP2A | DNA topoisomerase 2-alpha | 0.039515271 | -1.436542183 | down |
| CUX1 | Homeobox protein cut-like | 0.040335839 | 0.686333149 | up |
| ZNF431 | Zinc finger protein 431 (Fragment) | 0.04209632 | -1.797140555 | down |
| EML5 | Echinoderm microtubule-associated protein-like 5 | 0.042360121 | -10.51005226 | down |
| SPATS2L | SPATS2-like protein | 0.04387229 | 0.692563207 | up |
| RAB1A | Ras-related protein Rab-1A | 0.045177154 | -1.522547619 | down |
| G3BP1 | G3BP stress granule assembly factor 1 | 0.046196568 | -8.319714668 | down |
| SPSB4 | SplA/ryanodine receptor domain and SOCS box containing 4 (Fragment) | 0.049154821 | 5.110844384 | up |
| ZSCAN26 | Zinc finger and SCAN domain containing 26 | 0.049629196 | -2.149707594 | down |

**Supplementary Table 3.** All DEPs observed in Monastrol-treated PC-3 cells

(compared to the negative control).

| Gene Name | Description | pValue | logFC | Status |
| --- | --- | --- | --- | --- |
| SND1 | Staphylococcal nuclease domain-containing protein | 0.0000345 | -3.258727314 | down |
| UBA6 | Ubiquitin-like modifier-activating enzyme 6 | 0.0000474 | 1.904312037 | up |
| NR3C1 | Glucocorticoid receptor | 0.0000875 | 2.350783001 | up |
| ANXA6 | Annexin (Fragment) | 0.000216733 | -2.954351224 | down |
| DSTYK | Dual serine/threonine and tyrosine protein kinase isoform X7 | 0.000243602 | 1.253529179 | up |
| MMS19 | MMS19 nucleotide excision repair protein homolog | 0.000272529 | 2.926615688 | up |
| MTCL1 | Microtubule crosslinking factor 1 | 0.000312615 | -1.387332378 | down |
| MYO5A | Myosin VA | 0.000329089 | 1.710033952 | up |
| GAN | Epididymis secretory sperm binding protein (Fragment) | 0.000479828 | 0.889395607 | up |
| ZNF233 | Zinc finger protein 233 | 0.000635298 | -0.940864651 | down |
| NOMO3 | NODAL modulator 3 | 0.000816959 | -1.039155061 | down |
| DNAH14 | Dynein axonemal heavy chain 14 | 0.000853931 | -3.448094592 | down |
| MIA2 | MIA SH3 domain ER export factor 2 | 0.000869303 | 0.733295874 | up |
| NEBL | Nebulette variant 4 | 0.00091536 | 1.805223379 | up |
| PRKCB | Protein kinase C beta (Fragment) | 0.000979943 | -2.382742041 | down |
| MSH6 | DNA mismatch repair protein | 0.001104691 | -4.104017579 | down |
| COX2 | Cytochrome c oxidase subunit 2 | 0.001208432 | 1.958039807 | up |
| HLA-A | MHC class I antigen | 0.001237977 | -1.849943135 | down |
| PYCR2 | Pyrroline-5-carboxylate reductase 2 | 0.001257346 | -1.428695507 | down |
| GARRE1 | Granule associated Rac and RHOG effector 1 (Fragment) | 0.001281878 | 0.761690131 | up |
| CDK12 | Cyclin-dependent kinase 12 | 0.001296946 | -1.860751721 | down |
| MYH14 | Myosin-14 | 0.001300084 | -4.658535637 | down |
| FSTL5 | Follistatin-related protein 5 | 0.001405681 | -1.681597168 | down |
| TOGARAM1 | KIAA0423_ isoform CRA_a | 0.001429443 | -1.424028973 | down |
| PSMD2 | 26S proteasome non-ATPase regulatory subunit 2 | 0.00170025 | -1.045299606 | down |
| COL6A6 | Collagen alpha-6(VI) chain | 0.001874086 | -1.074074999 | down |
| MPDZ | Multiple PDZ domain protein | 0.002072836 | -1.10930415 | down |
| RPLP1 | Large ribosomal subunit protein P1 | 0.002151266 | -1.107501185 | down |
| SLC12A9 | Solute carrier family 12 member 9 (Fragment) | 0.002365311 | 1.967312842 | up |
| TUBA3C | Tubulin alpha-3C chain | 0.002404169 | -2.682123108 | down |
| PUF60 | Poly(U)-binding-splicing factor PUF60 | 0.00242812 | -1.520530074 | down |
| CDK13 | Cyclin dependent kinase 13 (Fragment) | 0.002479662 | -2.853958626 | down |
| FAN1 | Fanconi-associated nuclease | 0.00251904 | -1.087162743 | down |
| RAVER1 | Ribonucleoprotein PTB-binding 1 | 0.002576187 | -1.625294008 | down |
| HLA-B | MHC class I antigen (Fragment) | 0.00273178 | -1.208267845 | down |
| CACNB4 | Calcium voltage-gated channel auxiliary subunit beta 4 (Fragment) | 0.002823337 | 1.418517226 | up |
| PSMB6 | Proteasome subunit beta | 0.003060634 | -2.245425838 | down |
| CSF1R | Platelet-derived growth factor receptor-like protein (Fragment) | 0.003080242 | -1.778928289 | down |
| MPDU1 | Mannose-P-dolichol utilization defect 1_ isoform CRA_a | 0.003088702 | 6.565686686 | up |
| ARID1A | AT-rich interactive domain-containing protein 1A | 0.003217466 | 1.561708455 | up |
| NPIPB4 | Nuclear pore complex interacting protein family member B4 | 0.003387505 | 1.106786623 | up |
| YWHAQ | Tyrosine 3-monooxygenase/tryptophan 5-monooxygenase activation protein theta (Fragment) | 0.003459749 | -2.616161766 | down |
| PPA2 | Inorganic pyrophosphatase 2_ mitochondrial | 0.003624686 | -6.013740387 | down |
| ARHGAP21 | Rho GTPase activating protein 21 | 0.003843163 | -2.321175385 | down |
| TRIM28 | Transcription intermediary factor 1-beta | 0.004195105 | -0.952234518 | down |
| SHANK3 | SH3 and multiple ankyrin repeat domains 3 | 0.004203469 | 0.864600116 | up |
| ABCC12 | ATP binding cassette subfamily C member 12 | 0.004455573 | 0.982694918 | up |
| MEF2D | Myocyte-specific enhancer factor 2D | 0.004944646 | 1.32593789 | up |
| ANK3 | Ankyrin 3 (Fragment) | 0.005172021 | -1.708292526 | down |
| ZNF232 | Zinc finger protein 232 | 0.005295376 | -1.122418488 | down |
| HLA-B | MHC class I antigen | 0.005419612 | 2.91341843 | up |
| MGRN1 | E3 ubiquitin-protein ligase (Fragment) | 0.005976233 | -1.373139191 | down |
| U2AF2 | Splicing factor U2AF subunit | 0.006412307 | -1.265860081 | down |
| NONO | Non-POU domain containing octamer-binding isoform 1 (Fragment) | 0.00645931 | -2.261270206 | down |
| BAZ2B | Bromodomain adjacent to zinc finger domain protein 2B | 0.006526592 | -1.002064536 | down |
| DSG2 | Desmoglein-2 | 0.006612243 | -4.221138502 | down |
| AP3B1 | Adaptor-related protein complex 3 beta 1 subunit isoform 1 (Fragment) | 0.007065873 | -1.185511044 | down |
| HLA-DRB1 | MHC class II antigen (Fragment) | 0.007248457 | -2.183471777 | down |
| POLRMT | DNA-directed RNA polymerase_ mitochondrial | 0.007367754 | -1.25264558 | down |
| UBE2O | (E3-independent) E2 ubiquitin-conjugating enzyme | 0.007402928 | -1.613771895 | down |
| EEF1A2 | Elongation factor 1-alpha | 0.007409821 | -1.038143934 | down |
| GRIPAP1 | GRIP1 associated protein 1 | 0.007556529 | 1.321677629 | up |
| NCOA2 | Nuclear receptor coactivator 2 (Fragment) | 0.007690625 | 0.942934924 | up |
| UBE2D3 | Ubiquitin-conjugating enzyme E2 D3 | 0.007716486 | -1.491676486 | down |
| EML5 | Echinoderm microtubule-associated protein-like 5 | 0.007839705 | -1.11156912 | down |
| SAP130 | Sin3A associated protein 130 | 0.007892951 | -1.093059858 | down |
| CYFIP2 | Cytoplasmic FMR1-interacting protein | 0.007999972 | -1.185967974 | down |
| LMO7 | LIM domain 7 (Fragment) | 0.008054866 | 1.317398426 | up |
| USP43 | Ubiquitin carboxyl-terminal hydrolase 43 | 0.008099481 | -2.198030775 | down |
| SMC1A | Structural maintenance of chromosomes 1A | 0.008926369 | 1.000611035 | up |
| SPIN2A | Spindlin family member 2A | 0.009233296 | -1.577497285 | down |
| MIF | Macrophage migration inhibitory factor (Fragment) | 0.009296318 | -0.961284723 | down |
| SFN | 14-3-3 protein sigma | 0.009356522 | -1.101725891 | down |
| ERBB4 | Receptor tyrosine-protein kinase erbB-4 | 0.009508496 | -4.077712451 | down |
| DDHD1 | DDHD domain containing 1 (Fragment) | 0.009573098 | 0.613696672 | up |
| PLXNB2 | Plexin-B2 | 0.00957807 | -1.383715095 | down |
| HEL-S-284 | Aconitate hydratase_ mitochondrial | 0.01014179 | -2.106094314 | down |
| CHD3 | DNA helicase (Fragment) | 0.010598182 | -1.370600543 | down |
| MYLK | Myosin light chain kinase | 0.010814119 | 0.80036476 | up |
| CERK | Ceramide kinase | 0.010982836 | -1.45485006 | down |
| GAPVD1 | GTPase activating protein and VPS9 domains 1 | 0.011084989 | -1.107750885 | down |
| GON4L | GON4L protein (Fragment) | 0.011196187 | 1.347487998 | up |
| COBLL1 | Cordon-bleu WH2 repeat protein like 1 | 0.011474914 | -1.37113015 | down |
| HLA-DRB1 | MHC class II antigen (Fragment) | 0.011564826 | -1.547575261 | down |
| NPR2 | Guanylate cyclase | 0.011833454 | -1.178076973 | down |
| RAD23A | UV excision repair protein RAD23 homolog A | 0.01251338 | -2.628081917 | down |
| ARHGAP23 | ARHGAP23 (Fragment) | 0.014700494 | -1.118036298 | down |
| SATB2 | SATB homeobox 2 (Fragment) | 0.014880434 | 0.712307626 | up |
| RAB3GAP2 | RAB3 GTPase activating non-catalytic protein subunit 2 | 0.014943447 | -1.601821939 | down |
| TMEM132A | Transmembrane protein 132A | 0.015510041 | 7.005059769 | up |
| CTU2 | Cytoplasmic tRNA 2-thiolation protein 2 | 0.016317524 | -1.233779504 | down |
| ULK4 | Serine/threonine-protein kinase ULK4 | 0.016758442 | -1.635429601 | down |
| GRIA4 | Glutamate receptor | 0.017395525 | 1.506937826 | up |
| EPHA6 | Ephrin type-A receptor 6 | 0.01780984 | 0.793234753 | up |
| GUK1 | guanylate kinase | 0.018179653 | -1.11078008 | down |
| HLA-B | MHC class I antigen (Fragment) | 0.018458934 | -1.126056621 | down |
| NPR2 | Guanylate cyclase | 0.019167433 | 0.642083964 | up |
| ECT2 | Protein ECT2 | 0.019529921 | 0.88632392 | up |
| MAPK3 | mitogen-activated protein kinase | 0.020077324 | 1.00839003 | up |
| KEL | Kell blood group antigen | 0.020425426 | -1.525568311 | down |
| SCRIB | Protein scribble homolog | 0.022661266 | 1.536359295 | up |
| GAK | Cyclin G associated kinase | 0.023490893 | -1.15268819 | down |
| GREB1 | Protein GREB1 | 0.023641765 | -1.11095976 | down |
| CCDC39 | Coiled-coil domain-containing protein 39 | 0.027238236 | -3.264303155 | down |
| EIF2S3 | Eukaryotic translation initiation factor 2 subunit 3 | 0.029011291 | -1.569636636 | down |
| NPM1 | Nucleophosmin | 0.030243248 | -1.168709521 | down |
| PCDH19 | Protocadherin-19 | 0.031222368 | -3.281788251 | down |
| HLA-B | MHC class I antigen | 0.031267104 | -1.177656208 | down |
| ZNF516 | Zinc finger protein 516 | 0.031572552 | -1.128532762 | down |
| PCDH15 | Protocadherin related 15 | 0.033695184 | -3.861087385 | down |
| HEL-S-297 | SH3 domain-binding glutamic acid-rich-like protein 3 | 0.033832044 | -2.261240541 | down |
| HLA-DRB1 | MHC class II antigen (Fragment) | 0.03402699 | -2.412977231 | down |
| GBF1 | Golgi brefeldin A resistant guanine nucleotide exchange factor 1 | 0.035088084 | 2.815457341 | up |
| DDX11L8 | Putative ATP-dependent DNA helicase DDX11-like protein 8 | 0.037286284 | -1.500355533 | down |
| NEFH | Neurofilament heavy polypeptide | 0.038713306 | -0.969333557 | down |
| AR | Androgen receptor | 0.039148031 | -1.953512993 | down |
| ABCB4 | Phosphatidylcholine translocator ABCB4 | 0.040769604 | -1.831636777 | down |
| EHS-2 | EHS-2 protein (Fragment) | 0.043092575 | 2.286291477 | up |
| AMBRA1 | Autophagy and beclin 1 regulator 1 | 0.044646221 | 1.728562129 | up |
| SRP72 | SRP72 protein (Fragment) | 0.04527505 | -1.045318392 | down |
| RGS3 | Regulator of G protein signaling 3 | 0.0457724 | 4.253591531 | up |
| POGLUT3 | Protein O-glucosyltransferase 3 | 0.048630853 | -2.153784591 | down |
| KALRN | Kalirin RhoGEF kinase | 0.049887988 | -0.97329206 | down |

**Supplementary Table 4.** The 172 DEPs common to the PC-3 cells treated with (+)-PTC or Nocodazole (NOC).

| Gene Name | Description | pValue.NOC | logFC.NOC | pValue.PTC | logFC.PTC | stauts |
| --- | --- | --- | --- | --- | --- | --- |
| ABCB4 | Phosphatidylcholine translocator ABCB4 | 0.000679395 | -4.513870787 | 0.00867303 | -4.139601998 | down |
| ABL1 | Tyrosine-protein kinase ABL1 | 0.004437263 | -1.857030045 | 0.002261918 | -1.7774806 | down |
| ACTN4 | Alpha-actinin-4 (Fragment) | 0.00630321 | -1.723981831 | 0.01219901 | -1.666031858 | down |
| ANAPC7 | Anaphase-promoting complex subunit 7 | 0.014222222 | 5.370291315 | 0.003566738 | 5.67245772 | up |
| ANGPTL4 | Angiopoietin like 4 (Fragment) | 0.017142005 | -6.15111235 | 0.021617638 | -4.548126518 | down |
| ANK3 | Ankyrin 3 (Fragment) | 0.019434554 | -4.221193961 | 0.001280788 | -4.059302952 | down |
| ANKMY1 | Ankyrin repeat and MYND domain containing 1 | 0.013646156 | -3.886681763 | 0.002218278 | -3.191068742 | down |
| AOC3 | Amine oxidase [copper-containing] 3 | 0.001419173 | -1.80616764 | 0.008012 | -1.518836129 | down |
| AP2A1 | AP-2 complex subunit alpha-1 | 0.000495566 | -3.371039728 | 0.000122427 | -3.401883062 | down |
| AP2A2 | Adaptor related protein complex 2 subunit alpha 2 (Fragment) | 0.002613487 | -3.072518371 | 0.000432391 | -3.03690165 | down |
| APC2 | Adenomatous polyposis coli protein 2 | 0.025369356 | -2.908448894 | 0.006850556 | -3.269363776 | down |
| ARHGAP21 | Rho GTPase activating protein 21 | 0.000875246 | -7.097374511 | 0.000646939 | -4.651014796 | down |
| ARID1A | AT-rich interactive domain-containing protein 1A | 0.002307617 | 1.639049606 | 0.001592638 | 2.696512973 | up |
| ARID4B | AT-rich interactive domain-containing protein 4B | 0.000396545 | 3.730328908 | 0.000553309 | 3.299765048 | up |
| BAZ2A | Bromodomain adjacent to zinc finger domain 2A | 0.006378241 | -1.874491694 | 0.01550101 | -2.011569234 | down |
| BAZ2B | Bromodomain adjacent to zinc finger domain protein 2B | 0.000759975 | -1.784315238 | 0.001784936 | -1.97636206 | down |
| CACNB4 | Calcium voltage-gated channel auxiliary subunit beta 4 (Fragment) | 0.003646116 | 1.458342852 | 0.011623896 | 1.123919262 | up |
| CBL | E3 ubiquitin-protein ligase CBL | 0.026129693 | -3.33005737 | 0.001154237 | -3.317029265 | down |
| CDHR1 | Cadherin-related family member 1 | 0.00144004 | -6.547756676 | 0.049114071 | -3.148141789 | down |
| CDK12 | Cyclin-dependent kinase 12 | 9.95E-05 | -3.869952664 | 0.001045965 | -3.084067594 | down |
| CDK13 | Cyclin dependent kinase 13 (Fragment) | 0.01583194 | -3.115856893 | 0.003804024 | -3.062479842 | down |
| CFAP44 | Cilia- and flagella-associated protein 44 | 0.005831933 | 1.566500149 | 0.002236399 | 2.028809884 | up |
| CFL1 | Cofilin-1 | 0.001984473 | -1.820838316 | 0.007996817 | -1.676912933 | down |
| CFL2 | Cofilin-2 | 0.001230163 | -1.722421901 | 0.011327161 | -1.420296726 | down |
| CHCHD2 | Coiled-coil-helix-coiled-coil-helix domain-containing protein 2 | 9.63E-05 | -4.593661719 | 0.001623714 | -4.366908954 | down |
| CIT | non-specific serine/threonine protein kinase (Fragment) | 0.006540815 | 1.779876732 | 0.00206488 | 1.406808992 | up |
| CLEC7A | C-type lectin domain family 7 member A isoform 1 (Fragment) | 0.000338165 | 2.936787355 | 0.000609758 | 2.375726739 | up |
| COL16A1 | Collagen alpha-1(XVI) chain | 0.007261864 | 3.602934184 | 0.002645833 | 3.343536839 | up |
| COL4A1 | Collagen alpha-1(IV) chain | 0.001739454 | -3.361250142 | 0.012023185 | -3.025209968 | down |
| COL6A6 | Collagen alpha-6(VI) chain | 0.000715791 | -3.000550049 | 0.002960869 | -2.547999987 | down |
| COX2 | Cytochrome c oxidase subunit 2 | 0.007049114 | 0.678042724 | 0.024532329 | 0.72594262 | up |
| CSF1R | Platelet-derived growth factor receptor-like protein (Fragment) | 0.014743809 | -4.085447509 | 6.75E-06 | -3.801365695 | down |
| CTNND2 | Catenin delta 2 | 0.001310079 | -1.342258874 | 0.018294298 | -1.352839 | down |
| CTU2 | Cytoplasmic tRNA 2-thiolation protein 2 | 0.025903222 | -6.410810675 | 0.000783129 | -5.427182399 | down |
| CUX1 | Homeobox protein cut-like | 0.040335839 | 0.686333149 | 0.008299561 | 0.618614975 | up |
| DCDC1 | Doublecortin domain containing 1 | 0.005445086 | -3.647936919 | 0.02304881 | -3.09211686 | down |
| DDX11L8 | Putative ATP-dependent DNA helicase DDX11-like protein 8 | 0.006814442 | -6.424670284 | 0.000205105 | -6.64744264 | down |
| DDX46 | RNA helicase | 0.002212798 | -2.17090118 | 0.006331327 | -1.857792653 | down |
| DMPK | non-specific serine/threonine protein kinase | 0.026727489 | -3.823099074 | 0.02167626 | -3.805805207 | down |
| DNMT1 | DNA (cytosine-5)-methyltransferase | 0.027997793 | 0.744638902 | 0.010297237 | 0.755171362 | up |
| DOCK2 | DOCK2 (Fragment) | 0.007944625 | 1.504020181 | 0.008712563 | 1.307265279 | up |
| DOCK7 | Dedicator of cytokinesis protein 7 | 0.024665628 | -1.949460325 | 0.015220045 | -1.63548715 | down |
| DSG2 | Desmoglein-2 | 0.001101012 | -4.874372389 | 0.005296667 | -5.086609016 | down |
| EEF1A2 | Elongation factor 1-alpha | 0.033680883 | -1.638953374 | 0.02781941 | -1.342917806 | down |
| EIF2S3 | Eukaryotic translation initiation factor 2 subunit 3 | 0.010411092 | -2.904503787 | 0.004321867 | -3.04903478 | down |
| EIF3S9 | Eukaryotic translation initiation factor 3 subunit B | 0.001817321 | 2.464459396 | 0.003254645 | 1.457473197 | up |
| EIF4A2 | Eukaryotic initiation factor 4A-II | 0.028909374 | -1.506241473 | 0.044345868 | -1.808615072 | down |
| EML5 | Echinoderm microtubule-associated protein-like 5 | 0.042360121 | -10.51005226 | 9.95E-05 | -5.006406227 | down |
| ENO1 | Alpha-enolase | 0.008529153 | -1.510734832 | 0.013593186 | -1.479286572 | down |
| EPHA6 | Ephrin type-A receptor 6 | 0.008564538 | 0.981570783 | 0.006327993 | 1.093520244 | up |
| ERVK-21 | Endogenous retrovirus group K member 21 Env polyprotein | 0.016474637 | 1.372611974 | 0.014282932 | 1.598938827 | up |
| FAM21A | FAM21A protein | 0.017756773 | 1.809899602 | 0.014987217 | 2.80575458 | up |
| FAN1 | Fanconi-associated nuclease | 0.000213901 | -4.226101141 | 0.000547665 | -4.411569708 | down |
| FLJ00119 | Epididymis secretory sperm binding protein (Fragment) | 0.004505431 | -2.214378407 | 0.009963654 | -2.081870044 | down |
| FLT1 | Vascular endothelial growth factor receptor 1 | 0.001030441 | 1.146884861 | 0.002828837 | 1.097800436 | up |
| FSHD | Facioscapulohumeral muscular dystrophy | 0.015717348 | -5.120624589 | 5.60E-05 | -5.34690162 | down |
| FSTL5 | Follistatin-related protein 5 | 0.002397851 | -4.018402008 | 0.005981852 | -3.38899082 | down |
| FUT6 | 4-galactosyl-N-acetylglucosaminide 3-alpha-L-fucosyltransferase FUT6 | 0.024245432 | -5.188661708 | 0.011304037 | -3.221125732 | down |
| G3BP2 | Ras GTPase-activating protein-binding protein 2 | 0.019324935 | -2.22141551 | 0.001307843 | -1.975263823 | down |
| GAPVD1 | GTPase activating protein and VPS9 domains 1 | 0.008395816 | -2.592866204 | 0.004472951 | -1.805024333 | down |
| GARRE1 | Granule associated Rac and RHOG effector 1 (Fragment) | 0.038873137 | 0.712457145 | 0.01851565 | 0.985052683 | up |
| GART | Trifunctional purine biosynthetic protein adenosine-3 | 0.001426175 | -2.027744803 | 0.002575672 | -1.646639445 | down |
| GON4L | GON4L protein (Fragment) | 0.004822189 | 1.180470398 | 0.003501594 | 1.826325498 | up |
| GPR85 | G protein-coupled receptor 85 | 0.004582168 | 2.421774623 | 0.006943082 | 2.122453697 | up |
| GREB1 | Protein GREB1 | 0.00072745 | 3.19140897 | 0.005413376 | 1.579806733 | up |
| GRID2 | Glutamate ionotropic receptor delta type subunit 2 (Fragment) | 0.003596187 | -3.664672913 | 0.018306185 | -2.388865057 | down |
| GUK1 | guanylate kinase | 0.005025594 | -3.598364034 | 0.020777233 | -3.381584428 | down |
| H2AX | Histone H2AX | 0.000986091 | -3.90962729 | 0.001313946 | -3.103315658 | down |
| HEATR1 | HEAT repeat-containing protein 1 | 0.034211309 | -3.02865452 | 0.005936325 | -3.04074738 | down |
| HLA-A | MHC class I antigen | 0.000376301 | -3.799106589 | 0.000139707 | -2.521602429 | down |
| HLA-DQB1 | MHC class II antigen (Fragment) | 0.019984208 | -1.540081393 | 0.019694046 | -1.657598031 | down |
| HNRNPDL | Heterogeneous nuclear ribonucleoprotein D like | 0.018353287 | -3.662644426 | 0.000535177 | -4.302995778 | down |
| HNRNPU | Heterogeneous nuclear ribonucleoprotein U | 0.013649385 | -1.638297973 | 0.039860655 | -1.307745228 | down |
| HNRPA3 | Epididymis secretory sperm binding protein | 0.008041398 | 2.875472814 | 0.007778685 | 2.537104058 | up |
| IFT172 | Intraflagellar transport 172 | 0.005186156 | -1.982298149 | 0.004383754 | -1.54233516 | down |
| KAT8 | Histone acetyltransferase KAT8 | 0.010353085 | -1.510564531 | 0.008050151 | -1.394841361 | down |
| KCNA10 | Potassium voltage-gated channel subfamily A member 10 | 0.008710447 | 2.467629115 | 0.004155831 | 2.153975468 | up |
| KDM2B | Lysine-specific demethylase 2B | 0.000145761 | -10.48859341 | 0.022930191 | -9.069387934 | down |
| KIF5A | Kinesin family member 5A | 0.013579048 | -4.300807744 | 0.017176443 | -4.231251037 | down |
| KRT16 | Keratin_ type I cytoskeletal 16 | 0.013792614 | -2.936575606 | 0.017878224 | -2.384705489 | down |
| KRT40 | Keratin 40 | 0.016644483 | -2.213542119 | 0.047713764 | -1.948039236 | down |
| LCORL | Ligand dependent nuclear receptor corepressor like | 0.014547101 | 1.366842565 | 0.036267197 | 1.536886446 | up |
| LDHA | L-lactate dehydrogenase A chain | 0.006190101 | -1.796414823 | 0.002375742 | -1.575047843 | down |
| MAP1B | MAP1B protein (Fragment) | 0.009669532 | -2.263101485 | 0.015522007 | -1.744978135 | down |
| MAPK4 | mitogen-activated protein kinase | 0.012982445 | -1.904390729 | 0.015798452 | -1.385082235 | down |
| MATR3 | Matrin-3 | 0.00854201 | -1.464885496 | 0.028812221 | -1.28770654 | down |
| MCM7 | DNA replication licensing factor MCM7 | 0.022568042 | -2.557168283 | 0.020686196 | -2.935743303 | down |
| MEI1 | Meiosis inhibitor protein 1 | 0.000535985 | -1.814860502 | 0.039183627 | -1.406718823 | down |
| MIA2 | MIA SH3 domain ER export factor 2 | 0.004176188 | -2.255197649 | 0.002591551 | -1.852853222 | down |
| MIF | Macrophage migration inhibitory factor (Fragment) | 0.001328428 | -3.167049873 | 0.011135618 | -2.435097807 | down |
| MMS19 | MMS19 nucleotide excision repair protein homolog | 0.00055795 | 2.345215463 | 0.001968511 | 2.1090133 | up |
| MPDZ | Multiple PDZ domain protein | 0.011132632 | -3.39612566 | 0.038524572 | -2.790925113 | down |
| MPRIP | Myosin phosphatase Rho interacting protein (Fragment) | 0.019637205 | 1.107858957 | 0.01265354 | 1.684967648 | up |
| MYH14 | Myosin-14 | 0.000840501 | -5.508872614 | 0.004678989 | -4.764344018 | down |
| MYH3 | Myosin-3 | 0.009118948 | -1.82508675 | 0.015673901 | -1.418676155 | down |
| MYLK | Myosin light chain kinase | 0.006166362 | 2.149872869 | 0.004038542 | 3.00072571 | up |
| MYO5A | Myosin VA | 0.000614223 | 1.07170899 | 0.003408143 | 1.14585381 | up |
| N4BP1 | NEDD4-binding protein 1 | 0.000676751 | 1.213129897 | 0.002969955 | 1.301210548 | up |
| NAV3 | Neuron navigator 3 | 0.001225634 | -3.229005662 | 0.032815027 | -3.020457262 | down |
| NCOA2 | Nuclear receptor coactivator 2 (Fragment) | 0.005813658 | -2.967191878 | 0.025062511 | -1.578247261 | down |
| NES | Nestin | 0.008908584 | 0.763052039 | 0.002941541 | 0.786524856 | up |
| NME1 | Nucleoside diphosphate kinase A | 0.001909211 | -3.800790883 | 0.000451617 | -3.754215984 | down |
| NME2 | Nucleoside diphosphate kinase B | 0.003607936 | -2.541330462 | 0.000473594 | -2.29344523 | down |
| NONO | Non-POU domain containing octamer-binding isoform 1 (Fragment) | 0.012792354 | -2.072786923 | 0.038053315 | -1.907675346 | down |
| NPEPPS | Aminopeptidase | 0.000376377 | -4.730181511 | 0.020381843 | -4.870282825 | down |
| NR3C1 | Glucocorticoid receptor | 0.005771456 | 1.631567693 | 0.000970388 | 1.502167908 | up |
| NRXN3 | Neurexin 3 | 0.015833215 | -1.744454087 | 0.024341364 | -1.677945075 | down |
| OAS1 | 2'-5'-oligoadenylate synthetase 1 | 0.007639158 | -3.974253817 | 0.005215305 | -3.156402539 | down |
| OR14J1 | Olfactory receptor | 0.007706106 | 0.986903313 | 0.009921688 | 0.600093945 | up |
| PABPC4 | Polyadenylate-binding protein | 0.001386545 | -4.839498145 | 0.024143181 | -3.749739224 | down |
| PAPLN | Papilin | 0.013721788 | 1.619871895 | 0.005468852 | 2.906920829 | up |
| PDE4A | 3'_5'-cyclic-AMP phosphodiesterase 4A | 0.010387959 | -4.103862564 | 0.007699696 | -4.298251384 | down |
| PDE8A | High affinity cAMP-specific and IBMX-insensitive 3'_5'-cyclic phosphodiesterase 8A | 0.006318631 | -2.009831585 | 0.010643051 | -1.813046437 | down |
| PFN1 | Profilin-1 | 0.002799204 | -1.343935032 | 0.003340978 | -1.371722972 | down |
| PGK1 | Phosphoglycerate kinase 1 | 0.001621271 | -1.32692256 | 0.006717774 | -1.314690544 | down |
| PHB | Prohibitin | 0.010844264 | -3.237390722 | 0.040315552 | -2.178823756 | down |
| PHIP | Pleckstrin homology domain interacting protein | 0.018578461 | 0.598966392 | 0.001889174 | 1.392892229 | up |
| PIK3CA | phosphatidylinositol-4_5-bisphosphate 3-kinase | 0.034419237 | 4.197691905 | 0.036882103 | 4.013366075 | up |
| POM121C | Nuclear envelope pore membrane protein POM 121C | 0.017393696 | 0.87333994 | 0.007973653 | 1.548863976 | up |
| POTEE | POTE ankyrin domain family member E | 0.003038191 | 0.911451545 | 0.006159249 | 0.806146952 | up |
| POTEF | POTE ankyrin domain family member F | 0.010011291 | -2.752802968 | 0.009004663 | -3.180729762 | down |
| PRDX5 | Peroxiredoxin-5_ mitochondrial | 0.005469789 | -2.601323 | 0.001907794 | -2.231444747 | down |
| PRKCB | Protein kinase C beta (Fragment) | 0.00474366 | -8.778161656 | 0.001142472 | -6.28544065 | down |
| PRX | Periaxin | 0.022598645 | 1.386619167 | 0.018485354 | 1.668490993 | up |
| PSMD2 | 26S proteasome non-ATPase regulatory subunit 2 | 0.020860034 | -1.929277834 | 0.004281034 | -2.285746373 | down |
| PSME2 | Proteasome activator complex subunit 2 | 0.001722775 | -6.120750101 | 0.003220078 | -3.666178014 | down |
| PTGES3 | Prostaglandin E synthase 3 | 0.012897883 | -1.840837033 | 0.009277911 | -1.982060709 | down |
| PTOV1 | Prostate tumor-overexpressed gene 1 protein | 0.005045589 | -7.023713687 | 0.006475824 | -8.861643593 | down |
| PTPRS | Receptor-type tyrosine-protein phosphatase S | 0.006626334 | 1.526182579 | 0.006700506 | 0.690749502 | up |
| PUF60 | Poly(U)-binding-splicing factor PUF60 | 0.000274243 | -4.301982009 | 0.007707506 | -3.212472482 | down |
| RAB3GAP2 | RAB3 GTPase activating non-catalytic protein subunit 2 | 0.010627069 | -4.077543555 | 0.01497852 | -3.20096964 | down |
| RAD23A | UV excision repair protein RAD23 homolog A | 0.004281041 | -3.510897623 | 0.001156839 | -4.150296127 | down |
| RAN | GTP-binding nuclear protein Ran | 0.029046467 | 1.476560717 | 0.035380348 | 1.371816004 | up |
| RAVER1 | Ribonucleoprotein PTB-binding 1 | 0.000841645 | -3.449002529 | 0.001946706 | -3.461933467 | down |
| RBM20 | RNA-binding protein 20 | 0.005500253 | 2.577186214 | 0.007309103 | 2.206418545 | up |
| RPL12 | Large ribosomal subunit protein uL11 | 0.001711772 | -2.915363817 | 0.000977498 | -2.867855566 | down |
| RPL22 | Large ribosomal subunit protein eL22 | 0.001422221 | -3.279533427 | 5.32E-05 | -2.821156235 | down |
| RPLP1 | Large ribosomal subunit protein P1 | 0.008008907 | -1.798316463 | 0.00472042 | -1.682915412 | down |
| RPLP2 | Large ribosomal subunit protein P2 | 0.005278267 | -1.886647476 | 0.010874151 | -1.732267154 | down |
| RPS5 | Small ribosomal subunit protein uS7 (Fragment) | 0.004049868 | -2.131955309 | 0.00492749 | -1.957040797 | down |
| RPS7 | 40S ribosomal protein S7 | 0.005728248 | -1.445223511 | 0.044146593 | -1.466664616 | down |
| RTTN | Rotatin (Fragment) | 0.002504443 | 1.41862779 | 0.001437504 | 1.143971106 | up |
| SCN4A | Sodium channel protein type 4 subunit alpha | 0.009161312 | 1.079175257 | 0.00196654 | 1.374226241 | up |
| SCRIB | Protein scribble homolog | 0.014723151 | 1.078576004 | 0.030382174 | 0.792881277 | up |
| SFN | 14-3-3 protein sigma | 0.004850294 | -6.68670609 | 0.002792903 | -4.986797639 | down |
| SIN3A | SIN3 transcription regulator family member A | 0.005494165 | -4.479093808 | 0.010353711 | -2.374059527 | down |
| SLC12A9 | Solute carrier family 12 member 9 (Fragment) | 0.004787844 | 1.488571104 | 0.003232538 | 1.628458881 | up |
| SPATS2L | SPATS2-like protein | 0.04387229 | 0.692563207 | 0.04151793 | 0.597627712 | up |
| SPTAN1 | Spectrin alpha_ non-erythrocytic 1 (Fragment) | 0.033762149 | 0.635977519 | 0.013692559 | 0.96721497 | up |
| SRP72 | SRP72 protein (Fragment) | 0.000550959 | -6.745367265 | 0.000930978 | -5.193310554 | down |
| TAB3 | TGF-beta-activated kinase 1 and MAP3K7-binding protein 3 | 0.009738698 | -7.562871364 | 0.003077034 | -5.028000027 | down |
| TCF12 | Transcription factor 12 (Fragment) | 0.002181393 | 1.88978162 | 0.001866382 | 1.433147736 | up |
| TENC1 | Tensin like C1 domain containing phosphatase (Tensin 2) | 0.009317613 | -6.644211454 | 0.02568547 | -3.535440325 | down |
| TNRC6A | Trinucleotide repeat containing adaptor 6A (Fragment) | 0.000114816 | -2.412822709 | 0.003027053 | -1.991331454 | down |
| TOGARAM1 | KIAA0423_ isoform CRA_a | 0.018001155 | -2.205159435 | 0.020998925 | -1.667788212 | down |
| TPP2 | Tripeptidyl-peptidase 2 (Fragment) | 0.013045454 | -2.270908295 | 0.010895852 | -2.582569838 | down |
| TTLL3 | Tubulin tyrosine ligase like 3 | 0.016619995 | 5.493289333 | 0.016539201 | 5.538039821 | up |
| TUBA1B | Tubulin alpha-1B chain | 0.00597548 | -1.964857645 | 0.021169258 | -1.681138214 | down |
| TUBA3C | Tubulin alpha-3C chain | 0.000312932 | -5.779010671 | 0.002114856 | -5.157363128 | down |
| TUBA4A | Tubulin alpha-4A chain | 0.005874313 | -1.729536649 | 0.009748902 | -1.587634577 | down |
| TUBB8B | Tubulin beta 8B | 0.032125824 | -1.432376328 | 0.042913172 | -1.342162534 | down |
| UBR3 | E3 ubiquitin-protein ligase UBR3 | 0.037317504 | 2.467596829 | 0.006936542 | 2.919870861 | up |
| YBX1 | YBX1 protein (Fragment) | 0.000632336 | -4.539102078 | 0.003414008 | -4.562664838 | down |
| YWHAE/FAM22A fusion | 14-3-3 protein epsilon (Fragment) | 0.021948876 | -1.727422445 | 0.022060407 | -1.6648752 | down |
| YWHAQ | Tyrosine 3-monooxygenase/tryptophan 5-monooxygenase activation protein theta (Fragment) | 0.000389078 | -8.040551814 | 0.00083249 | -7.798678692 | down |
| ZC3H13 | Zinc finger CCCH-type containing 13 | 0.030037347 | -2.384691437 | 0.002049669 | -2.45395575 | down |
| ZNF233 | Zinc finger protein 233 | 0.001727891 | -1.461452404 | 0.003977176 | -1.354129124 | down |
| ZNF431 | Zinc finger protein 431 (Fragment) | 0.04209632 | -1.797140555 | 0.033874525 | -1.849123418 | down |
| ZNF516 | Zinc finger protein 516 | 0.004990442 | -2.533828598 | 0.010604637 | -2.454563127 | down |
| ZNF582 | Zinc finger protein 582 | 0.000584989 | -2.3604081 | 0.020737304 | -2.088118721 | down |
| ZNF717 | Zinc finger protein 717 | 0.002118366 | 0.986511853 | 0.003799796 | 0.801699987 | up |
| ZSCAN26 | Zinc finger and SCAN domain containing 26 | 0.049629196 | -2.149707594 | 0.018309781 | -1.580616657 | down |

**Supplementary table 5.** List of DEPs up-regulated from (+)-PTC-treated PC-3 cells related to the protein class DNA-binding transcriptional factor.

| **(+)- PTC-treated PC3 cells** | | |
| --- | --- | --- |
| **Gene Name** | **Protein Class** | **Protein Description** |
| LCORL | DNA-binding transcription facto | Ligand-dependent nuclear receptor corepressor-like protein |
| CUX1 | Homeodomain transcription factor | Homeobox protein cut-like 1 |
| NR3C1 | C4 zinc finger nuclear receptor | Glucocorticoid receptor |
| TCF12 | Basic helix-loop-helix transcription factor | Transcription factor 12 |
| ZNF717 | C2H2 zinc finger transcription factor | Zinc finger protein 717 |

**Supplementary table 6.** List of DEPs down-regulated from (+)-PTC-treated PC-3cells related to the protein classes actin or actin-binding cytoskeletal protein and microtubule or microtubule-binding cytoskeletal protein.

| **(+)- PTC-treated PC3 cells** | | |
| --- | --- | --- |
| **Category: actin or actin-binding cytoskeletal protein** | | |
| **Gene Name** | **Protein Class** | **Protein Description** |
| CFL1 | Non-motor actin binding protein (PC00165) | Cofilin-1 |
| CFL2 | Non-motor actin binding protein (PC00165) | Cofilin-2 |
| POTEF | Actin and actin-related protein (PC00039) | POTE ankyrin domain family member F |
| FAN1 | Non-motor actin binding protein (PC00165) | Fascin |
| MYH3 | Actin or actin-binding cytoskeletal protein (PC00041) | Myosin-3 |
| ACTN4 | Actin or actin-binding cytoskeletal protein (PC00041) | Alpha-actinin-4 |
| MYH14 | Actin or actin-binding cytoskeletal protein (PC00041) | Myosin-14 |
| PFN1 | Non-motor actin binding protein (PC00165) | Profilin-1 |
| **Category: microtubule or microtubule-binding cytoskeletal protein** | | |
| **Gene Name** | **Protein function** | **Protein Description** |
| TUBA4A | Tubulin (PC00228) | Tubulin alpha-4A chain |
| TUBA1B | Tubulin (PC00228) | Tubulin alpha-1B chain |
| TUBA3C | Tubulin (PC00228) | Tubulin alpha-3C chain |
| KIF5A | Microtubule binding motor protein (PC00156) | Kinesin heavy chain isoform 5A |
| TUBB8B | Tubulin (PC00228) | Tubulin beta 8B |
| EML5 | Microtubule or microtubule-binding cytoskeletal protein (PC00157) | Echinoderm microtubule-associated protein-like 5 |
| TOGARAM1 | Non-motor microtubule binding protein (PC00166) | TOG array regulator of axonemal microtubules protein 1 |
| MAP1B | Non-motor microtubule binding protein (PC00166) | Microtubule-associated protein 1B |

**Supplementary table 7*.*** The top five most significant enriched Reactome pathways associated with DEPs identified in Nocodazole-treated PC-3 cells (relative to the negative control group).

| **Pathway name** | **p-value** |
| --- | --- |
| **Upregulated protein** | |
| Nuclear receptor transcription pathway | 5.42^-8^ |
| RAC1 GTPases cycle | 1.68^-5^ |
| RHO GTPase cycle | 1.31^-4^ |
| RAC2 GTPases cycle | 3.18^-4^ |
| RHOB GTPase cycle | 9.8^-4^ |
| **Downregulated protein** |  |
| Microtubule-dependent trafficking of connexons from Golgi to the plasma membrane | 3.45^-7^ |
| Transport of connexons to the plasma membrane | 4.46^-7^ |
| RNA Polymerase I Promoter Opening | 6.73^-6^ |
| Carboxyterminal post-translational modifications of tubulin | 8.66^-6^ |
| PRC2 methylates histones and DNA | 1.56^-5^ |


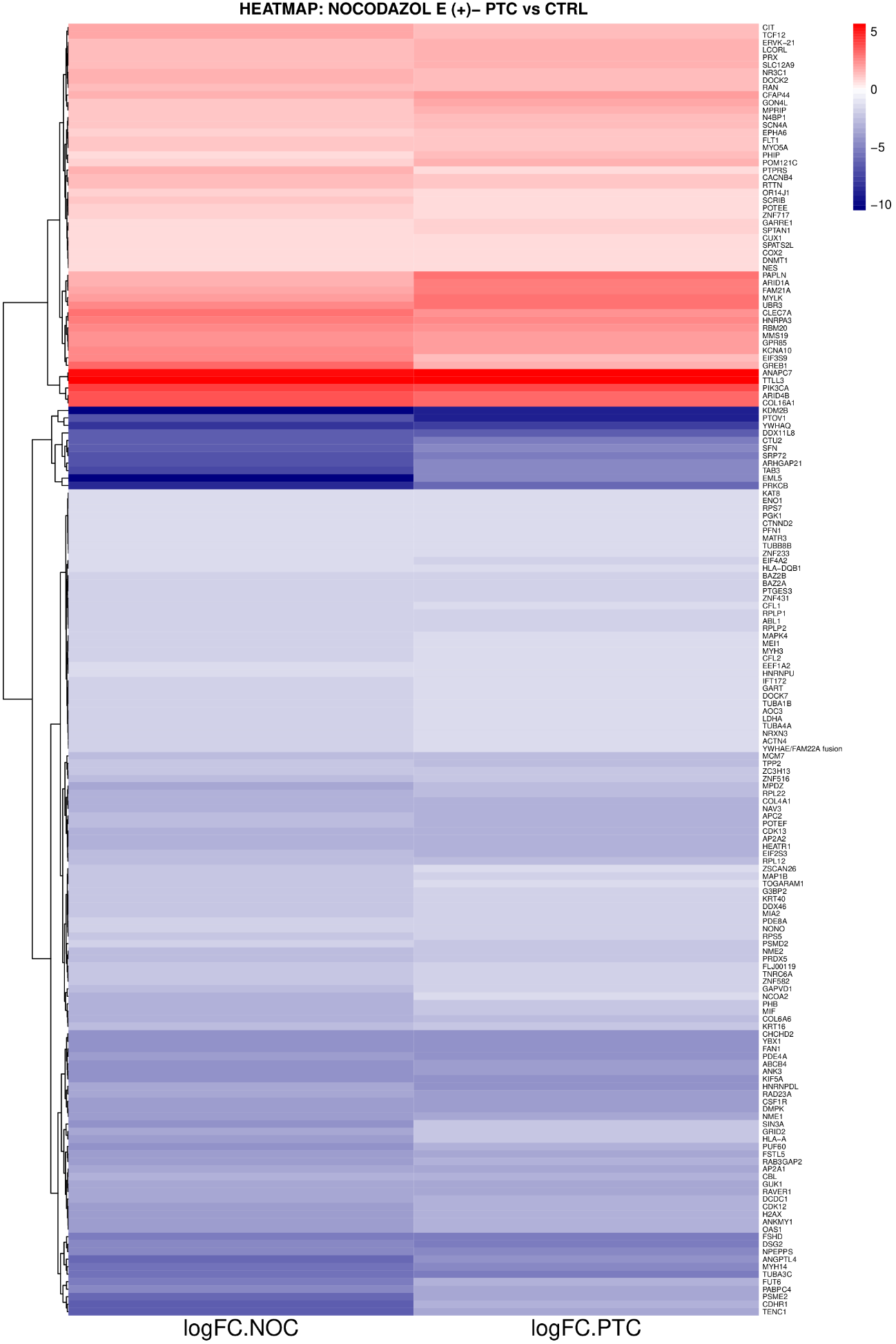


**Supplementary Figure 1.** Proteomic profile analyses of DEP among different treatments. Heatmap of common DEP between (+)- PTC and Nocodazole in PC3 cells. Scale color: Red (Up-regulated); Blue (Down-regulated) NOC: Nocodazole; PTC: (+)- PTC.


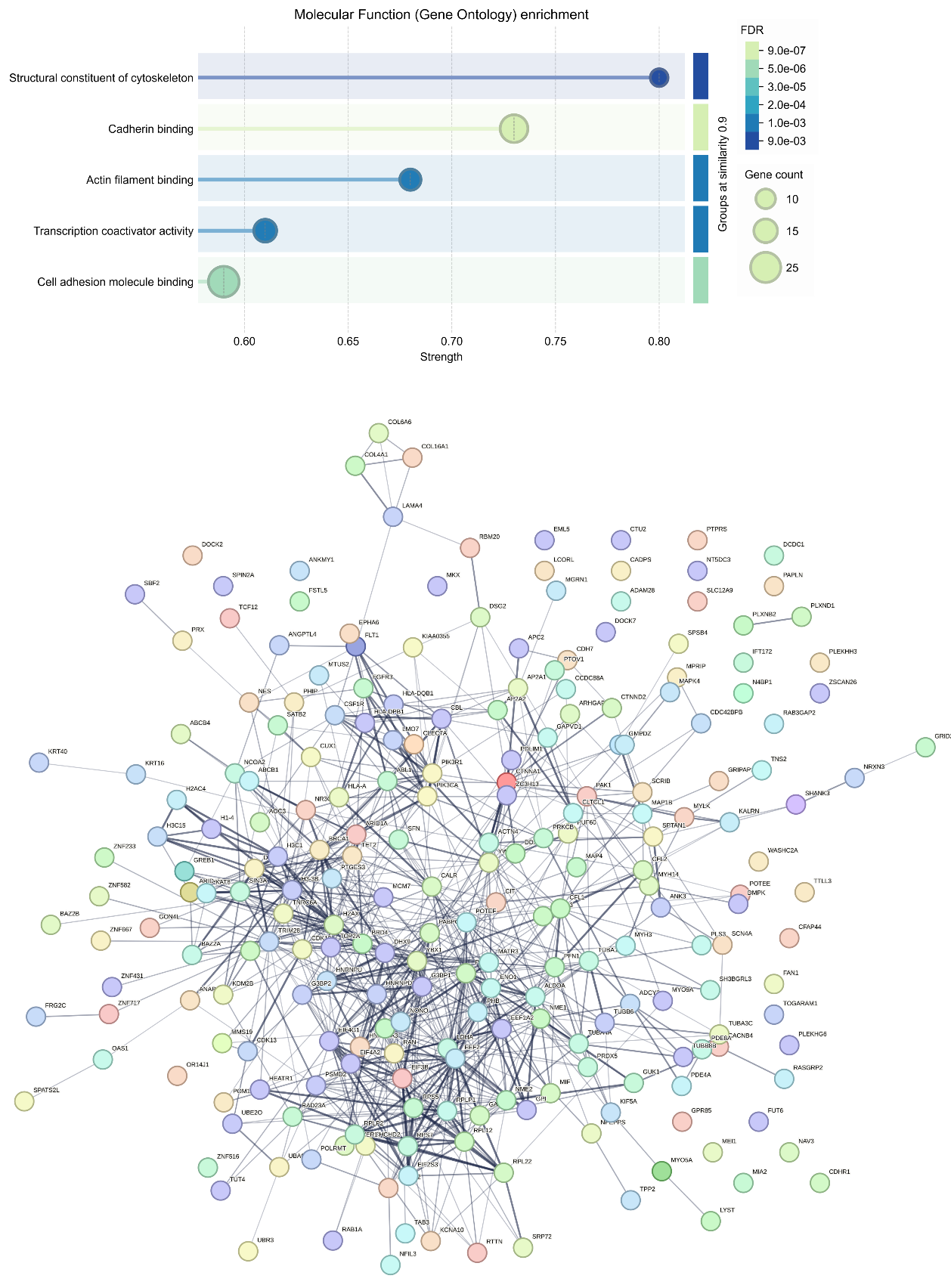


B

A

**Supplementary Figure 2.** Proteomic profile and Gene ontology enriched terms for DEP after treatment with Nocodazole. (A) GO functional enrichment analysis for Molecular function of DEP. (B) Protein-protein interaction network of DEP.


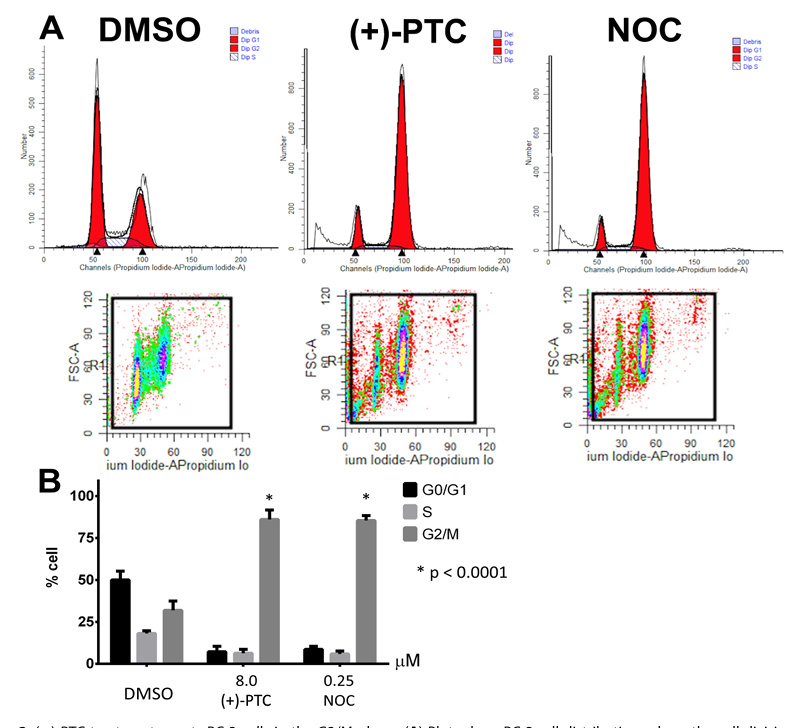


**Supplementary Figure 3.** (+)-PTC treatment arrests PC-3 cells in the G2/M-phase. (A) Plots show PC-3 cell distributions along the cell division cycle as determined by flow cytometry using Triton X-100 permeabilization and propidium iodide staining after 24 h incubation. Negative control cells were treated with the vehicle (DMSO) not exceeding 0.075%. NOC (0.25 μM) was used as a positive control. (B) Graphical representation of cytometry quantification results from non-synchronized cells showing subpopulations in the G0/G1-, S- and G2/M-phases of the cell cycle. When treated with 8.0 μM (+)-PTC, cells accumulated in the G2/M-phase and so mimicking the effects of NOC. NOC blocks cell-cycle progression by depolymerizing of MTs. Ten thousand events were acquired in each experiment. Data are derived from three independent experiments, each performed in quadruplicate. Two-way ANOVA then Bonferroni's tests were applied to the data. The asterisks indicate differences at the 95% significance levels (P<0.05). Figure from MORAES DE FARIAS; ROSA‐RIBEIRO; SOUZA; KOBARG et al., 2022, doi.org/10.1002/cbdv.202200102.


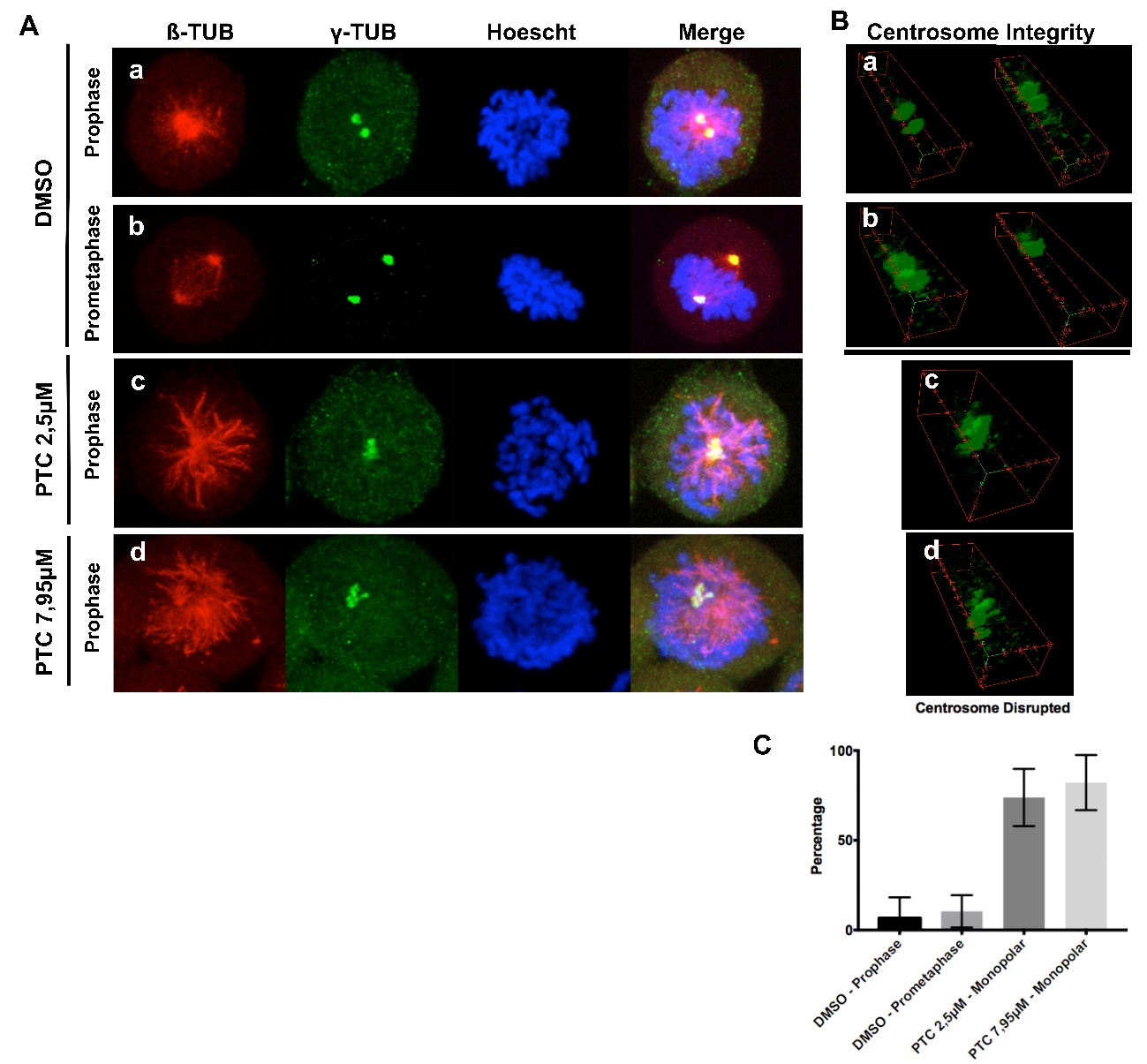


**Supplementary Figure 4.**  (+)-PTC induces monastral spindles in PC-3 cells. Cells were cultured for 24 h in the presence of vehicle (1% DMSO), (+)-PTC (2.5 μM or 8.0 μM), monastrol (50 μM, MON) or nocodazole (0.25 μM, NOC) and then fixed and stained with antibodies against β-tubulin (red), γ-tubulin (green) and pericentrin (magenta). DNA was counter-stained blue using Hoechst 33258. The images were collected in Z-stacks and converted to maximum intensity projections. All cells were analyzed and characterized using the same threshold value. (A) Representative images were obtained for the control and (+)-PTC-treated cells. (B) Representative images were obtained for the control and (+)-PTC-treated cells of the centrossome. (C) Quantitative results. Control PC-3 cells show typical prophase (A'a) and metaphase features including the presence of bipolar spindles (A’b). Monoastral spindles surrounded by rings of chromosomes were observed in the (+)-PTC-treated PC-3 cells (A’c (A’d). In (+)-PTC treated cells, over 80% of the mitosis had monastral (monopolar) spindles or NOC-induced multipolar spindles. At least 100 cells were examined and counted in each experiment– these conditions were repeated three times. Two-way ANOVA then Bonferroni's test was applied to the data. The asterisks indicate differences at the 95% significance levels (P<0.05). Figure from MORAES DE FARIAS; ROSA‐RIBEIRO; SOUZA; KOBARG et al., 2022, doi.org/10.1002/cbdv.202200102.


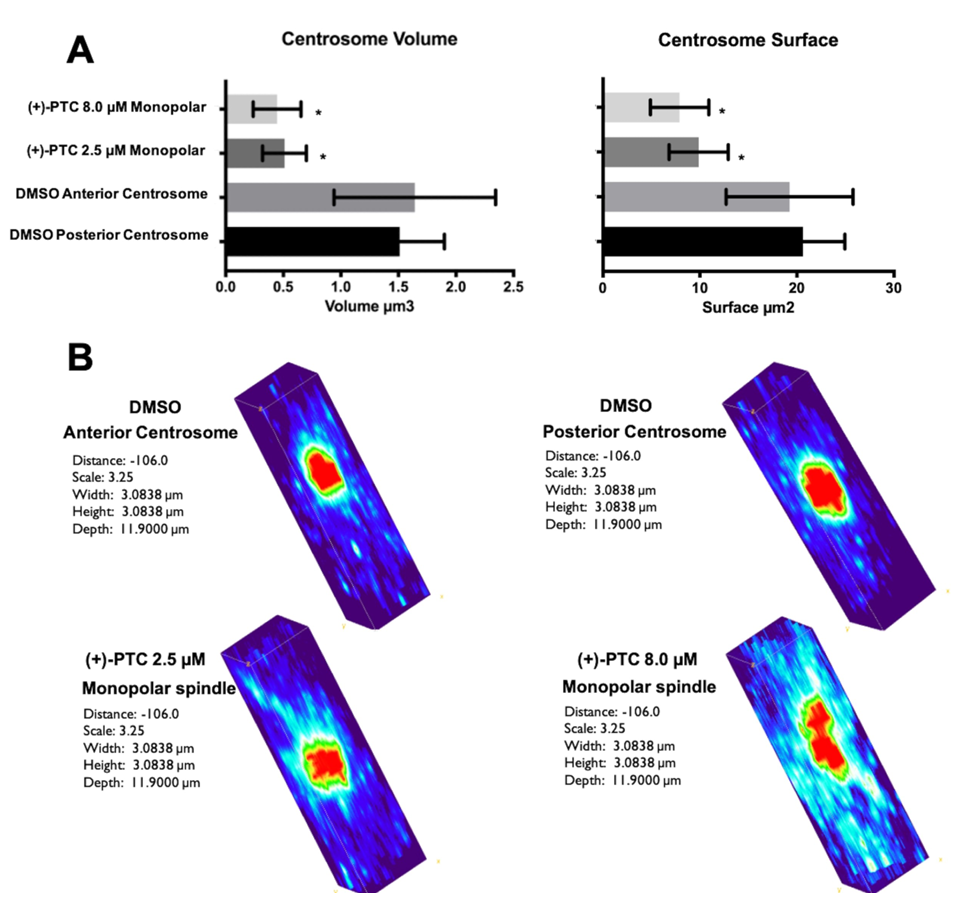


**Supplementary Figure 5.** (+)-PTC disrupts centrosome assembly in PC-3 cells. (A) The volumes and surface areas of the centrosomes were measured using the 3D Objects Counter plug-in. (B) Three-dimensional images of the volumes and surfaces of the anterior and posterior centrosomes showing the monopolar spindles after (+)-PTC treatment. (+)-PTC treatment caused volume and surface area reductions after 24 h. In untreated cells (DMSO alone) the anterior and posterior centrosomes were resolved while in those treated with (+)-PTC monoasters were in evidence and centrosome integrity as well as the resolution of its anterior and posterior components were compromised. All cells were analyzed and characterized using the same threshold value. The immunofluor escence intensities were obtained from thermal LUTs in a Max Projection of 0.1 μm Z-stacks, using the Image J Fiji software. Thirty cells were analyzed in each experiment. Two-way ANOVA then Bonferroni's tests were applied to the data. The asterisks indicate differences at the 95% significance levels (P<0.05). Figure from MORAES DE FARIAS; ROSA‐RIBEIRO; SOUZA; KOBARG et al., 2022, doi.org/10.1002/cbdv.202200102.


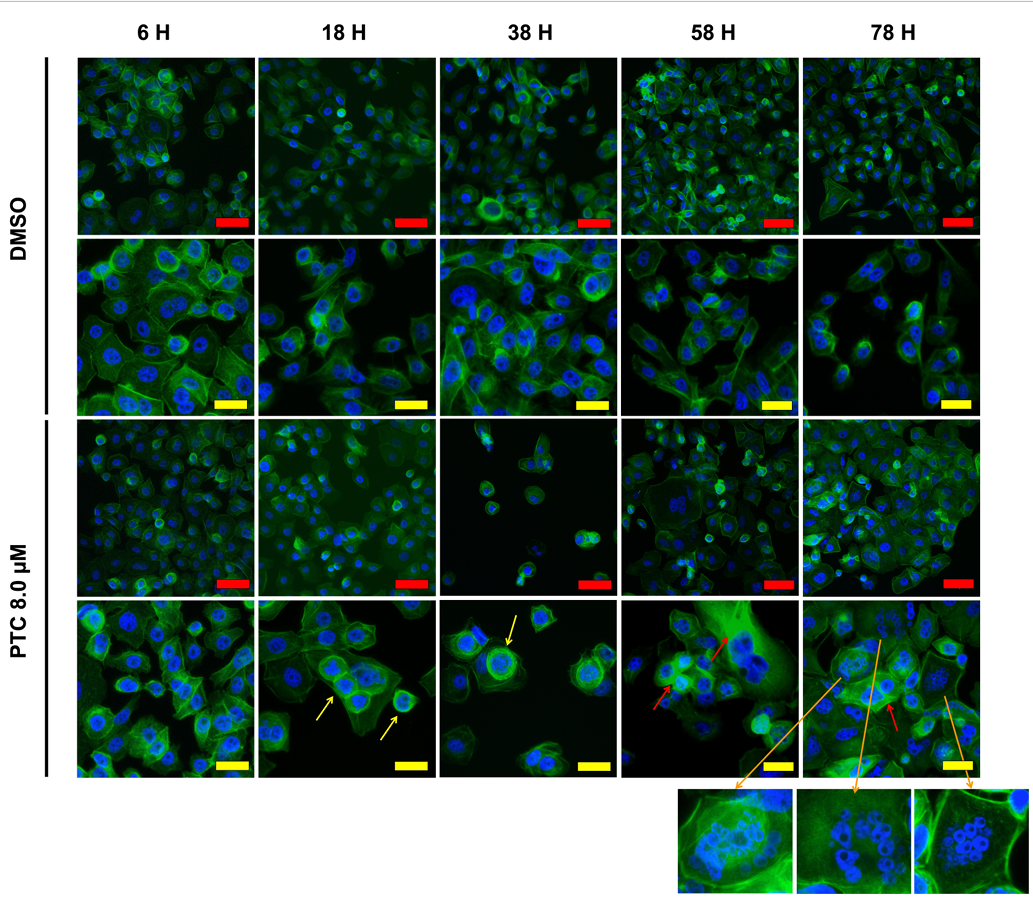


**Supplementary Figure 6.** (+)-PTC (8.0 μM) promotes late changes in cell morphology and actin distribution. Cells were exposed to (+)-PTC or vehicle (DMSO) and examined at 6, 18, 38, 58 and 78 h. Cells were fixed, stained and then analyzed by confocal microscopy. 6 h after exposure to (+)-PTC, there was no significant change in PC-3 cell morphology, as compared to the controls. 18 h after exposure the cell population was largely composed of mitotic cells (yellow arrows). After 38 h, the cell population had visibly diminished by comparison with the control cells. After 58 h, multinucleated cells and/or fragmented nuclei were seen (orange arrows) with the actin cytoskeleton now arranged in thick bundles around the cell nucleus (red arrows). These features remained 78 h after initial exposure to (+)-PTC (red scale bar=50 μm and the yellow scale bar=25 μm) Figure from MORAES DE FARIAS; ROSA‐RIBEIRO; SOUZA; KOBARG et al., 2022, doi.org/10.1002/cbdv.202200102.


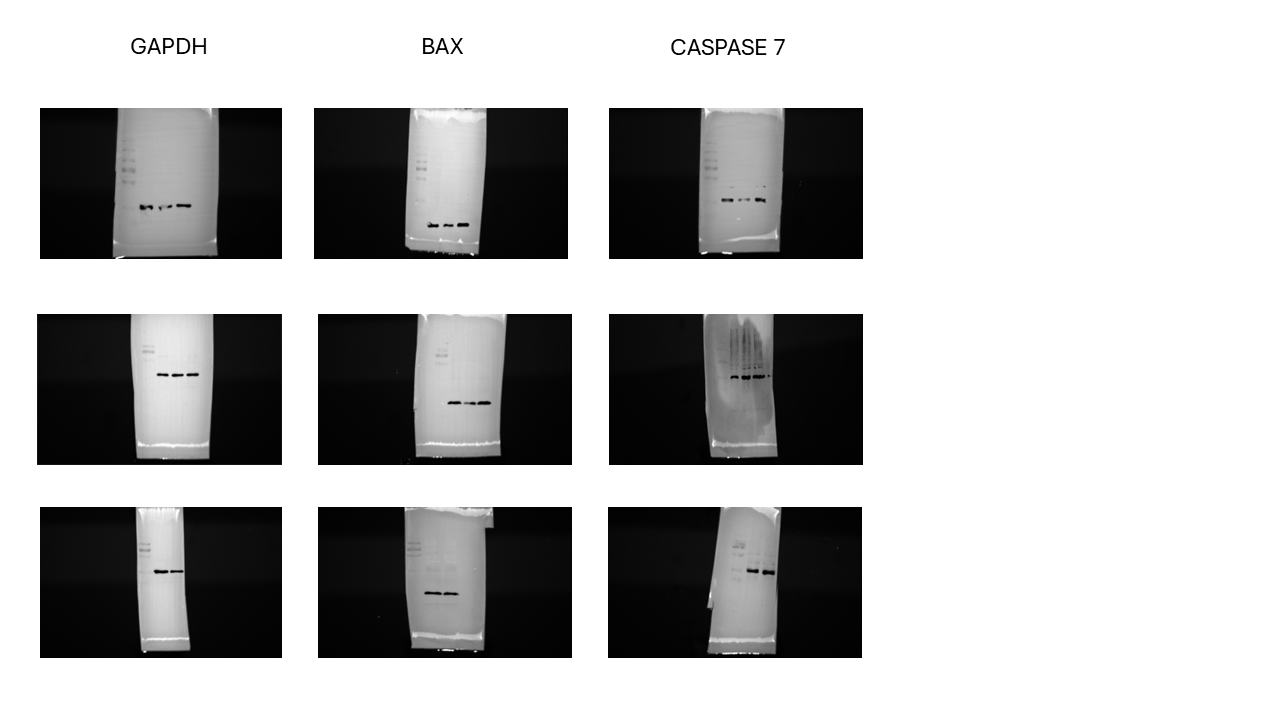


**Supplementary Figure 7.** Shows the whole blot after cutting membrane. Original cropped membranes for PC3 cells treated with (+)-PTC.
